# Tumor-Treating Fields Plus Temozolomide Versus Temozolomide Alone in Newly Diagnosed Glioblastoma: A Systematic Review and Bayesian Meta-Analysis with Meta-Regression

**DOI:** 10.3390/cancers18121947

**Published:** 2026-06-16

**Authors:** Plamen Penchev, Daniela Milanova-Ilieva, Lyubomir Gaydarski, Jorge Eduardo Alonso-Vera, Stefan Stavrevski, Petar-Preslav Petrov, Laurens Noack, Nikolai Ramadanov, Bogdana Suchorska

**Affiliations:** 1 Faculty of Medicine, Medical University of Plovdiv, 4001 Plovdiv, Bulgaria; 2 Department of Pediatrics, St. George’s University Hospital, 4002 Plovdiv, Bulgaria; 3 Department of Anatomy, Histology and Embryology, Medical University of Sofia, 1431 Sofia, Bulgaria; 4 Faculty of Medicine, Universidad Catolica de Santiago de Guayaquil, Guayaquil 090615, Ecuador; 5 Clinic of Neurosurgery, University Hospital “St. Marina”, 9010 Varna, Bulgaria; 6 Faculty of Medicine, Medical University of Varna, 9002 Varna, Bulgaria; 7 Department of Anatomy, Histology, and Embryology, Medical University of Plovdiv, 4002 Plovdiv, Bulgaria; 8 Center of Orthopaedics and Traumatology, Brandenburg Medical School, University Hospital Brandenburg, 14770 Brandenburg an der Havel, Germany; 9 Faculty of Health Science Brandenburg, Brandenburg Medical School Theodor Fontane, 16816 Brandenburg an der Havel, Germany; 10 Department of Neurosurgery, Heidelberg University Hospital, 69120 Heidelberg, Germany

**Keywords:** glioblastoma, tumor-treating fields, temozolomide, overall survival, progression-free survival

## Abstract

**Introduction:** Glioblastoma is the most aggressive primary brain tumor in adults, with limited survival despite multimodal therapy. Tumor-treating fields (TTFs) combined with temozolomide (TMZ) have emerged as a therapeutic option for newly diagnosed glioblastoma (nGBM), yet the magnitude, robustness, and generalizability of its benefit remain uncertain. This Bayesian meta-analysis aimed to determine whether the addition of TTFs to TMZ improves survival outcomes and safety profiles in patients with nGBM. **Methods:** We systematically searched PubMed, Scopus, and the Cochrane Library from inception to 5 April 2026, for studies comparing TTFs + TMZ with TMZ alone in nGBM (PROSPERO “CRD420261374813”). Outcomes included overall survival (OS; hazard ratio [HR]), progression-free survival (PFS; HR), and dermatologic adverse effects (proportions). A Bayesian random-effects model with vague priors for overall effects and informative/weakly informative priors for between-study heterogeneity was applied. Sensitivity analyses with alternative prior specifications were performed, and posterior probabilities (PPs), 95% credible intervals (CrIs), and posterior predictive probabilities (PPPs) were estimated. **Results:** Twelve studies (one randomized controlled trial and 11 observational studies) comprising 2797 patients were included (TTFs + TMZ: 1228; TMZ: 1569). TTFs + TMZ was associated with improved OS (HR 0.68; 95% CrI 0.57–0.80; 99% PP HR < 1) with a 96% PPP that future studies would also favor TTFs + TMZ. PFS was likewise improved (HR 0.67; 95% CrI 0.58–0.77; 99% PP HR < 1), with a 98% PPP favoring TTFs + TMZ in future studies. Subgroup analyses suggested that risk of bias contributed more to heterogeneity than study design. Among patients receiving TTFs + TMZ, the estimated proportion of dermatologic adverse effects was 57.8% (95% CrI 0.36–0.79), with high PPs of exceeding clinically meaningful thresholds. **Conclusions:** In this Bayesian meta-analysis, TTFs combined with TMZ was associated with improved OS and PFS in nGBM, although dermatologic toxicity was frequent. These findings support TTFs + TMZ as an effective adjunctive strategy, while emphasizing the importance of patient selection, adherence, and toxicity management.

## Simple Summary

Glioblastoma remains the most aggressive primary brain tumor in adults, with poor survival despite standard multimodal treatment. Tumor-treating fields (TTFs) have been introduced as a novel, non-invasive therapy used alongside temozolomide (TMZ), but their overall benefit has been debated. In this Bayesian meta-analysis, we combined evidence from clinical studies to evaluate whether adding TTFs to TMZ improves patient outcomes. We found that this combination is associated with a consistent and highly probable improvement in both overall and progression-free survival, with strong likelihood that future studies will confirm these benefits. Although skin-related side effects were common, they were generally localized and manageable. These findings support the use of TTFs as an effective addition to standard therapy while emphasizing the need for careful patient selection, adherence, and toxicity management in clinical practice.

## 1. Introduction

Glioblastoma (GBM) is the most aggressive and most common malignant primary brain tumor in adults, characterized by diffuse infiltration, marked intratumoral heterogeneity, rapid progression, and near-universal recurrence [1,2,3]. Despite advances in neurosurgical techniques, radiotherapy delivery, and systemic therapy, the prognosis of patients with newly diagnosed glioblastoma (nGBM) remains poor [2,4]. The current standard of care is the Stupp protocol, which consists of maximal safe resection followed by radiotherapy with concomitant and adjuvant temozolomide (TMZ), yet survival gains achieved with this multimodal approach remain limited [4,5]. TMZ, an oral alkylating agent that induces DNA damage and tumor cell apoptosis, represented a major therapeutic advance when incorporated into first-line treatment; however, even with this regimen, long-term survival remains uncommon [4,5,6]. This persistent therapeutic plateau has driven interest in adjunctive strategies capable of improving outcomes without substantially increasing systemic toxicity.

Tumor-treating fields (TTFs) have emerged as a novel, non-invasive antimitotic therapy for nGBM [7,8]. Delivered through transducer arrays applied to the scalp, TTFs generate low-intensity, intermediate-frequency alternating electric fields that disrupt mitotic spindle formation, interfere with chromosome segregation, and impair cytokinesis in rapidly dividing tumor cells [7,8]. In clinical practice, TTFs are delivered through a portable device connected to adhesive transducer arrays placed on the shaved scalp according to individualized tumor-location mapping. Treatment is generally intended for prolonged daily use during the maintenance phase of TMZ, and adherence is considered an important determinant of benefit. The technique is non-invasive and does not usually produce systemic toxicity, but it imposes practical burdens, including continuous device wear, scalp shaving, battery or power supply management, regular array replacement, skin care, and lifestyle adjustments. In addition, the cost of TTFs and reimbursement availability vary substantially across healthcare systems, which may influence patient access and real-world implementation [7,8].

The addition of TTFs to maintenance TMZ has been supported by randomized evidence and subsequently adopted in clinical practice in selected settings [8,9]. Nevertheless, important uncertainties remain. Although available studies suggest a survival benefit with TTFs plus TMZ, the magnitude and consistency of this effect across different clinical settings are still debated. The debate largely reflects differences in study design, patient selection, adherence to TTFs, and real-world implementation. The pivotal randomized trial reported improved survival with TTFs added to maintenance TMZ; however, the open-label nature of the trial, the dependence of benefit on device adherence, and differences between trial populations and routine clinical practice have led to ongoing discussion regarding generalizability. Subsequent observational studies have provided supportive real-world evidence, but these studies are inherently susceptible to residual confounding, treatment-selection bias, and variability in supportive care. Therefore, a synthesis that explicitly explores uncertainty, study design, and risk of bias may help clarify the consistency of the observed survival benefit [9].

In particular, the evidence base includes both randomized and observational studies, raising questions regarding heterogeneity, external validity, adherence-dependent effectiveness, and the influence of methodological bias [8,9,10]. In addition, while TTFs are generally not associated with systemic toxicities, dermatologic adverse effects are frequent and may affect treatment adherence and tolerability [11,12].

Previous evidence syntheses in oncology have predominantly relied on frequentist meta-analytic methods. Although widely used, these approaches are inherently limited in handling the uncertainty and complexity of clinical data, particularly when the number of studies is modest, study designs are mixed, and between-study heterogeneity is substantial [13,14]. Frequentist methods generally focus on point estimates and confidence intervals, which may provide less intuitive clinical interpretation and may not fully reflect uncertainty in the true effect distribution across studies. By contrast, Bayesian meta-analysis provides a robust framework for evidence synthesis under such conditions. It allows incorporation of prior distributions, direct probabilistic interpretation of treatment effects, and estimation of credible intervals and posterior predictive probabilities, thereby offering more clinically meaningful inference [13,14]. Moreover, Bayesian meta-regression enables exploration of sources of heterogeneity, such as study design and risk of bias, which is especially relevant in the evaluation of TTFs [14].

Accordingly, we conducted a systematic review and Bayesian meta-analysis with meta-regression to compare TTFs plus TMZ versus TMZ alone in patients with nGBM. By integrating available randomized and observational evidence within a Bayesian framework, this study aims to clarify the survival benefit, characterize the dermatologic toxicity burden, explore sources of heterogeneity, and provide predictive estimates that may better inform clinical decision-making and future research.

## 2. Methods

### 2.1. Protocol and Reporting Standards

This systematic review and meta-analysis followed the Cochrane Handbook for Systematic Reviews of Interventions and the Preferred Reporting Items for Systematic Reviews and Meta-Analysis Statement [15,16]. This meta-analysis did not require Institutional Review Board approval because it used data from previously published and publicly available articles. This systematic review and meta-analysis was registered with the International Prospective Register of Systematic Reviews (PROSPERO) under the ID “CRD420261374813”.

### 2.2. Eligibility Criteria

Studies that met all the following criteria were included in the meta-analysis: (1) adults (≥18 years) with newly diagnosed glioblastoma (nGBM), confirmed histopathologically, before first disease recurrence or progression; (2) patients must have completed the standard Stupp protocol (maximal safe resection followed by radiotherapy with concurrent TMZ); (3) patients must have undergone adjuvant TTFs, delivered through scalp transducer arrays as a non-invasive alternating electric-field therapy, in combination with adjuvant TMZ following completion of the Stupp protocol; (4) adjuvant TMZ alone as a control group following completion of chemoradiation; (5) studies must report at least one of the following outcomes of interest: overall survival (OS), progression-free survival (PFS), or dermatologic adverse effects; studies were not required to report all outcomes to be eligible; however, each study contributed only to the specific outcome meta-analysis for which extractable data were available; thus, studies reporting OS but not PFS were included only in the OS analysis, and studies reporting dermatologic adverse effects were included only in the adverse-event proportion analysis; (6) randomized controlled trials (RCTs) and observational studies (prospective or retrospective). Studies were excluded if they met one of the following criteria: (1) pediatric populations (<18 years) or mixed populations without GBM-specific data; (2) studies evaluating recurrent GBM, progressive GBM, mixed newly diagnosed and recurrent GBM cohorts without separately extractable nGBM data, or studies in which newly diagnosed status could not be confirmed; (3) studies evaluating TTFs without concurrent adjuvant TMZ, or other non-comparable interventions; (4) studies where the control group does not receive adjuvant TMZ alone after the Stupp protocol (e.g., only RT + concurrent TMZ, TTFs only, or other chemotherapy regimens); (5) studies not reporting OS, PFS or dermatologic adverse effects, or with insufficient extractable data; (6) case reports, case series, narrative reviews, systematic reviews, meta-analyses, conference abstracts, editorials, letters, expert opinions, the gray literature, or dissertations; (7) overlapping patient population.

### 2.3. Search Strategy and Data Extraction

We systematically searched PubMed, Scopus, and Cochrane CENTRAL from inception to 1 April 2026 with the following search strategy: (glioblastoma OR GB OR GBM OR “glioblastoma multiforme” OR “grade IV astrocytoma” OR “grade 4 astrocytoma” OR “high grade glioma” OR “newly diagnosed GBM” OR “newly diagnosed GB” OR ndGBM OR ndGB) AND (“tumor treating fields” OR TTF OR TTFields OR NovoTTF-100A OR NovoTTF-200A OR Optune OR “TTF plus temozolomide” OR “TTFields plus Temozolomide” OR “tumor treating fields combined with temozolomide” OR “tumor treating fields plus temozolomide” OR “TTF combined with temozolomide”) AND (temozolomide OR TMZ OR “TMZ alone” OR “temozolomide alone” OR “maintenance treatment” OR “standard therapy” OR chemotherapy OR “temozolomide chemotherapy” OR “no TTFields” OR “no TTFields group” OR “without TTFields”). The search strategy was structured according to the PICO framework, combining population terms for glioblastoma/nGBM, intervention terms for TTFs/TTFields, and comparator or treatment-context terms for TMZ-based standard therapy. Broad TMZ-related terms were intentionally included to maximize sensitivity, whereas final eligibility was determined during screening according to the predefined requirement for TTFs + TMZ versus TMZ alone after completion of standard chemoradiation. Restrictions were limited to articles published in English, and the gray literature was excluded. In addition, the reference lists and citations of all included studies were manually screened to identify any further relevant articles. Two authors (P.P. and D.M.) independently extracted the data according to predefined criteria and quality assessment methods, using Rayyan software [17]. Any disagreements were resolved through consensus.

### 2.4. Outcomes

The meta-analysis assessed OS and PFS as primary outcomes, while dermatologic adverse events were evaluated as secondary outcomes. In addition, subgroup analyses were performed for each endpoint, stratified by study design and study-level risk of bias, to reduce potential selection bias.

### 2.5. Quality Assessment

The risk of bias (ROB) of the RCTs was assessed using the Cochrane Collaboration’s RoB 2.0 tool [18], which evaluates bias across five domains: the randomization process, deviations from intended interventions, missing outcome data, measurement of outcomes, and selection of the reported result. Each study was classified as having low risk, some concerns, or a high risk of bias. The ROB of observational studies was evaluated using ROBINS-I [19], which categorizes bias as low, moderate, serious, or critical. Two authors (E.A. and S.S.) independently conducted the assessments, with disagreements resolved by consensus. Publication bias was assessed using contour-enhanced funnel plots with the trim-and-fill method, plotting individual study weights against effect estimates. In accordance with Cochrane guidelines, the Egger test was performed for OS and PFS because more than 10 studies were included in the meta-analysis [16].

### 2.6. Bayesian Analysis

We fitted a random-effects meta-analysis model within a Bayesian framework, in which prior assumptions are updated using the observed data to generate a posterior distribution [20]. For the primary analysis, vague priors were used for the overall treatment effect across all analyses, while an informative prior was specified for the between-study heterogeneity parameter. Sensitivity analyses were also performed using alternative prior specifications to determine whether the choice of priors meaningfully influenced the results or conclusions. In Bayesian inference, the posterior distribution reflects the updated probability distribution of the treatment effect after integrating prior assumptions with the observed study data. Further details regarding the model structure and prior distributions are provided in the Supplementary Content.

Bayesian random-effects meta-analysis produces marginal posterior distributions for both the overall effect and between-study heterogeneity. These distributions were summarized using the mean and 95% highest-density intervals, hereafter referred to as credible intervals (CrIs), defined as the narrowest interval containing 95% of the posterior probability density [21]. The primary estimands were reported as hazard ratios (HRs) and proportions, with additional emphasis placed on the estimation of posterior probabilities.

#### 2.6.1. Between-Study Heterogeneity and Prediction

By considering both within-study and between-study variability, random-effects meta-analysis provides a more appropriate framework for capturing overall heterogeneity [20,22]. Within the Bayesian framework, prior uncertainty regarding the heterogeneity parameter is explicitly incorporated, allowing estimation of the posterior distribution of between-study heterogeneity.

Moreover, the posterior predictive distribution enables a more comprehensive assessment of the potential influence of between-study heterogeneity. This distribution supports inference about what may be expected in a new study population that is exchangeable with the studies included in the meta-analysis [23]. It is therefore important for estimating plausible values of the true treatment association in future settings. The posterior predictive distribution incorporates uncertainty in both the overall effect size and the between-study standard deviation, generating parameter estimates for a new trial population independently of sample size and other population-specific characteristics [24].

All Bayesian analyses, including posterior predictive analyses, were performed using the R package “bayesmeta” [25]. All statistical analyses were conducted in R, version 4.3.1 [26].

#### 2.6.2. Bayesian Meta-Analysis of Proportions

We pooled dermatologic adverse effects across studies using a binomial–normal (logistic random-effects) model. This model directly uses raw counts (events and total patients) and estimates study-specific proportions and an overall proportion through the Bayesian framework, which updates prior beliefs with the current data to form a posterior distribution [22].

We applied weakly informative priors for the overall proportion and between-study heterogeneity. We also fitted models with different priors in sensitivity analyses to check whether this choice of priors meaningfully impacted our results or conclusions. Full details about our model and priors can be found in the Supplement.

Results are reported as proportions with 95% credible intervals (CrIs, highest-density intervals). We also calculated posterior probabilities that the proportion of dermatologic adverse effects exceeded prespecified clinically meaningful thresholds of 30%, 50%, and 70%. These thresholds were selected to represent a common adverse-event burden, an adverse-event burden affecting approximately half of treated patients, and a high adverse-event burden, respectively. They were used to aid clinical interpretation rather than to define formal safety stopping boundaries.

The posterior predictive distribution was derived to describe the expected proportion in a future study.

Analyses were conducted using the “brms” R package [27] with the “cmdstanr backend” (Stan version 2.38.0) in R (R Environment version 4.4.3).

#### 2.6.3. Meta-Regression/Subgroup Analyses

We fitted Bayesian random-effects meta-regression meta-analysis models for subgroup analyses. We applied the “Intercept/offset” parametrization in the R package “bayesmeta” (http://doi.org/10.1016/j.cmpb.2022.107303). For primary analyses, a vague prior was set for the overall effect in the reference subgroup, a weakly informative prior for the subgroup difference parameter, and, for the between-study heterogeneity parameter, we applied similar priors used in the pairwise analyses. Sensitivity analyses with different priors for the between-study heterogeneity parameter were also conducted. Further details can be found in the Supplemental Content.

#### 2.6.4. Leave-One-Out (LOO) Analysis

We performed a leave-one-out sensitivity analysis, sequentially excluding one study at a time and re-fitting the Bayesian random-effects model. We applied the same priors in all models (primary and leave-one-out). This assessed the robustness of our conclusions to individual influential studies.

## 3. Results

### 3.1. Study Selection and Baseline Characteristics

**Figure 1:**
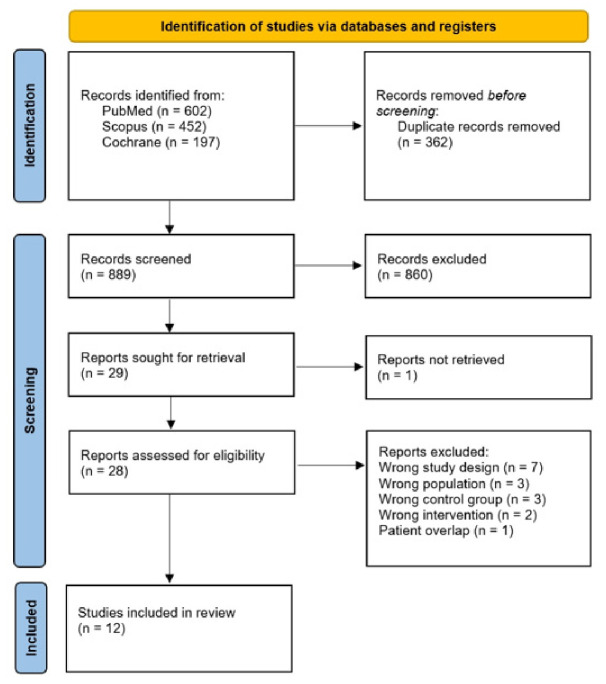
PRISMA flow chart and study selection.

The search strategy yielded a total of 1251 records. After removing duplicates and screening titles and abstracts, 29 studies underwent full-text review to assess eligibility according to the predefined inclusion and exclusion criteria (Figure 1). Twelve studies, including one randomized controlled trial and eleven observational studies, comprising a total of 2797 patients (TTFs + TMZ: 1228; TMZ: 1569), were included in the analysis [8,28,29,30,31,32,33,34,35,36,37,38]. Inter-rater agreement for study inclusion was high (Cohen’s κ = 0.82), indicating excellent consistency between reviewers. Disagreements were resolved by consensus. The mean age was 55.8 years in the TTFs + TMZ group and 57.7 years in the TMZ group, with females comprising 39.6% and 38.2% of patients, respectively. Baseline population characteristics are summarized in Table 1.

### 3.2. Pooled Analyses of the Included Studies

#### 3.2.1. OS

**Figure 2:**
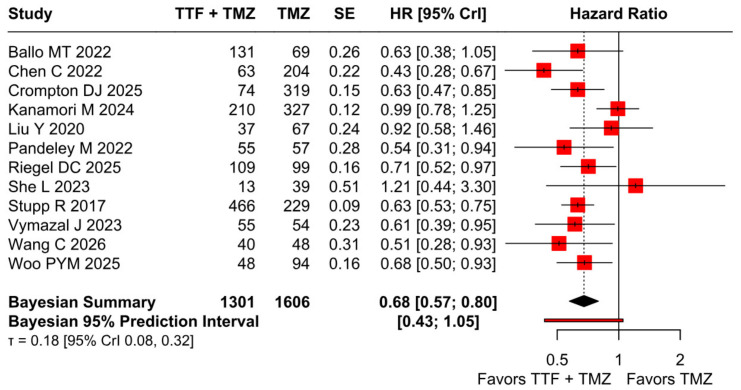
Forest plot of OS outcome. The pooled HR of 0.68 (95% CrI: 0.57 to 0.80) indicates a significant improvement in OS following the combination therapy. The PP of any survival benefit (HR < 1) was 99%, providing a high degree of certainty for a positive effect [8,28,29,30,31,32,33,34,35,36,37,38].

**Figure 3:**
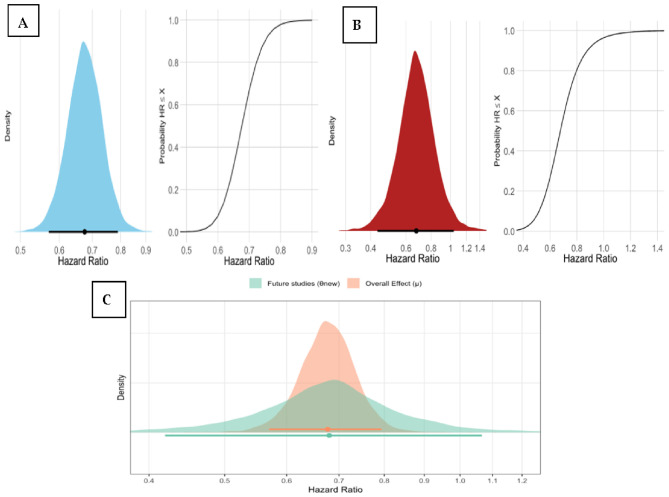
(**A**) Posterior distribution of the pooled hazard ratio (HR) for OS after TTFs + TMZ, with shaded areas indicating posterior probabilities across HR thresholds. (**B**) Posterior predictive distribution for OS, showing the expected range of effects in an exchangeable future study; the 95% predictive interval was 0.43–1.05, with a 96% probability of HR < 1. (**C**) Comparison of the current posterior and predictive distributions, showing wider uncertainty in the predictive distribution due to between-study heterogeneity.

A total of 12 studies were included in the meta-analysis of OS. The Bayesian random-effects meta-analysis yielded a posterior median HR of 0.68 (95% CrI: 0.57; 0.80) (Figure 2). The posterior median of the between-study heterogeneity (τ) was 0.18 (95% CrI: 0.08; 0.32). The posterior probability (PP) that TTFs + TMZ provided an OS benefit (HR < 1) compared to TMZ alone was 99%. Figure 3A shows the posterior distribution of the overall effect and posterior probabilities related to any HR cutoff. Regarding the predictive distribution, we found a 95% probability that the true HR of a future study would lie between 0.43 and 1.05 and 96% probability that it would be lower than 1 (Figure 3B). Figure 3C shows the comparison of current posterior estimates and future predictive distributions. Figure 3C demonstrates that the posterior distribution of the current pooled OS effect was narrower than the posterior predictive distribution. This indicates that, although the pooled evidence strongly favored TTFs + TMZ, the expected treatment effect in an exchangeable future setting may be more variable because the predictive distribution incorporates between-study heterogeneity in addition to uncertainty around the pooled effect.

**Figure 4:**
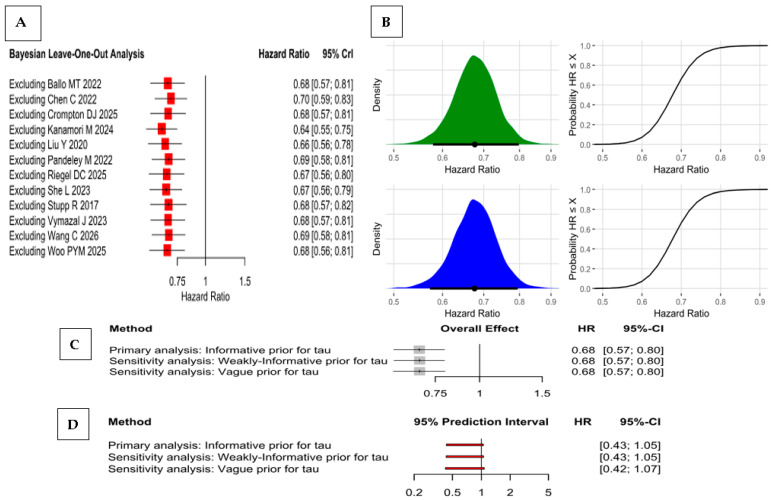
Robustness and prior-sensitivity analyses for OS [8,28,29,30,31,32,33,34,35,36,37,38]. (**A**) LOO analysis showing stable pooled HRs after sequential exclusion of individual studies. (**B**) Prior-sensitivity analysis showing stability of the posterior mean HR across different priors for between-study heterogeneity (τ). (**C**) Summary of posterior OS HR estimates under different heterogeneity priors. (**D**) Summary of posterior predictive distributions under different heterogeneity priors, illustrating the influence of τ prior choice on predictive uncertainty.

LOO sensitivity analysis confirmed the robustness of the primary findings for OS. Sequential omission of individual studies resulted in HR values ranging from 0.64 to 0.70, with all 95% CrIs remaining below one. No single study exerted a disproportionate influence on the overall estimate (Figure 4A). Sensitivity analyses with different priors demonstrated that the pooled effect estimates were robust to prior specification, though credible intervals were similar when using less informative (weakly informative and vague) priors (Figure 4B,C). Furthermore, the posterior predictive distributions did not exhibit higher sensitivity to the choice of the between-study heterogeneity prior, with weakly informative and vague priors resulting in similar prediction intervals (Figure 4D).

#### 3.2.2. PFS

**Figure 5:**
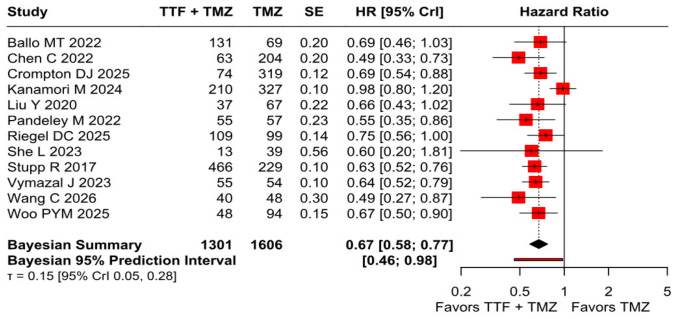
Forest plot of PFS outcome. The pooled HR of 0.67 (95% CrI: 0.58 to 0.77) indicates a significant improvement in PFS following the combination therapy. The PP of any survival benefit (HR < 1) was 99%, providing a high degree of certainty for a positive effect [8,28,29,30,31,32,33,34,35,36,37,38].

**Figure 6:**
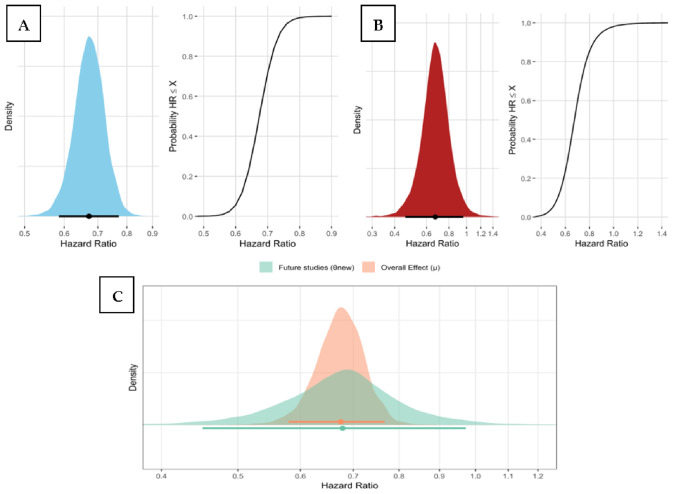
(**A**) Posterior distribution of the pooled HR for PFS after TTFs + TMZ, with shaded areas indicating posterior probabilities across HR thresholds. (**B**) Posterior predictive distribution for PFS, showing the expected range of effects in an exchangeable future study; the 95% predictive interval was 0.46–0.98, with a 98% probability of HR < 1. (**C**) Comparison of the current posterior and predictive distributions, showing wider uncertainty in the predictive distribution due to between-study heterogeneity.

A total of 12 studies were included in the meta-analysis of OS. The Bayesian random-effects meta-analysis yielded a posterior median HR of 0.67 (95% CrI: 0.58; 0.77) (Figure 5). The posterior median of the between-study heterogeneity (τ) was 0.15 (95% CrI: 0.05; 0.28). The PP that TTFs + TMZ provided an PFS benefit (HR < 1) compared to TMZ alone was 99%. Figure 6A shows the posterior distribution of the overall effect and posterior probabilities related to any HR cutoff. Regarding the predictive distribution, we found a 95% probability that the true HR of a future study would lie between 0.46 and 0.98 and 98% probability that it would be lower than 1 (Figure 6B). Figure 6C shows the comparison of current posterior estimates and future predictive distributions. Figure 6C similarly shows that the predictive distribution for PFS was wider than the current posterior distribution, reflecting the additional uncertainty expected when generalizing the pooled effect to an exchangeable future study population.

**Figure 7:**
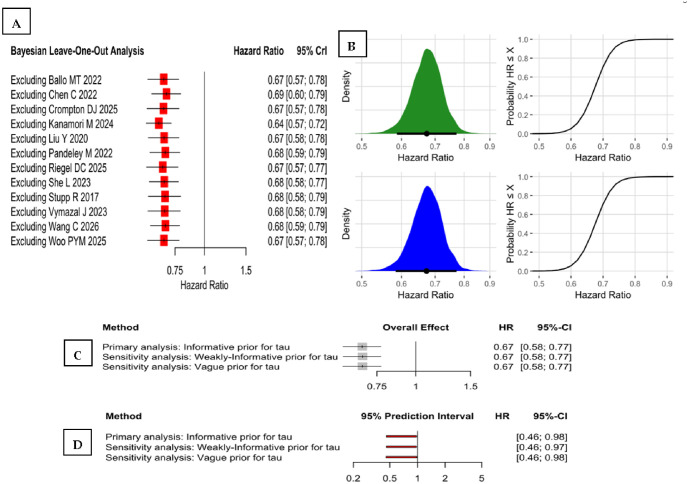
Robustness and prior-sensitivity analyses for PFS [8,28,29,30,31,32,33,34,35,36,37,38]. (**A**) LOO sensitivity analysis showing stable pooled HRs after sequential exclusion of individual studies. (**B**) Prior-sensitivity analysis showing stability of the posterior mean HR across different priors for between-study heterogeneity (τ). (**C**) Summary of posterior PFS HR estimates under different heterogeneity priors. (**D**) Summary of posterior predictive distributions under different heterogeneity priors, illustrating the influence of τ prior choice on predictive uncertainty.

LOO sensitivity analysis confirmed the robustness of the primary findings for PFS. Sequential omission of individual studies resulted in HR values ranging from 0.64 to 0.69, with all 95% CrIs remaining below one. No single study exerted a disproportionate influence on the overall estimate (Figure 7A). Sensitivity analyses with different priors demonstrated that the pooled effect estimates were robust to prior specification, and credible intervals were similar when using less informative (weakly informative and vague) priors (Figure 7B,C). Furthermore, the posterior predictive distributions exhibited higher sensitivity to the choice of the between-study heterogeneity prior, with weakly informative and vague priors resulting in similar prediction intervals (Figure 7D).

#### 3.2.3. Dermatologic Adverse Effects

**Figure 8:**
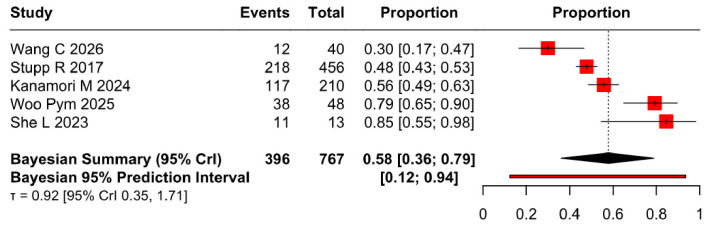
Posterior distribution of the pooled proportion from the Bayesian meta-analysis. The posterior median proportion is 0.58, with a 95% CrI ranging from 0.36 to 0.79, reflecting the uncertainty around the estimated overall effect [8,28,29,31,35].

**Figure 9:**
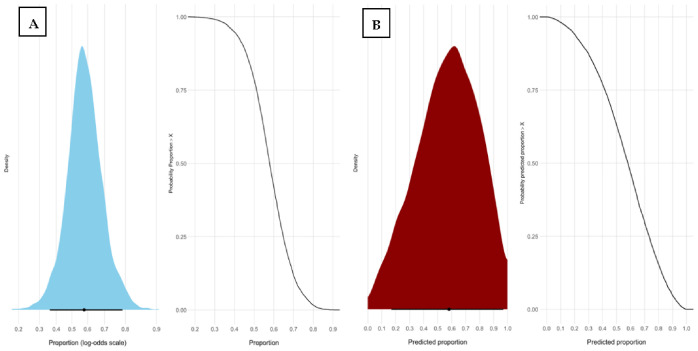
Posterior and posterior predictive distributions for dermatologic adverse effects. (**A**) Posterior distribution of the pooled proportion of dermatologic adverse effects after TTFs + TMZ, with shaded areas indicating posterior probabilities across proportion thresholds. (**B**) Posterior predictive distribution showing the expected range of adverse-effect rates in an exchangeable future study; the probabilities of exceeding 30%, 50%, and 70% were 87%, 63%, and 30%, respectively.

A total of five studies were included in the meta-analysis of dermatologic adverse effects. The Bayesian meta-analysis of proportions yielded a posterior median proportion of 0.58 (95% CrI 0.36; 0.79) (Figure 8). The posterior median of the between-study heterogeneity (τ, on the log-odds scale) was 0.92 (95% CrI 0.35; 1.71). The 95% prediction interval for the true proportion in a new study ranged from 0.12 to 0.94. Figure 9A shows the posterior distribution of the overall effect and posterior probabilities related to any proportion cutoff. The PPP that a future study would observe adverse-event rates exceeding 30%, 50%, and 70% was 87%, 63%, and 30%, respectively (Figure 9B). These findings suggest a high probability that adverse-event rates will exceed clinically meaningful thresholds in future studies.

**Figure 10:**
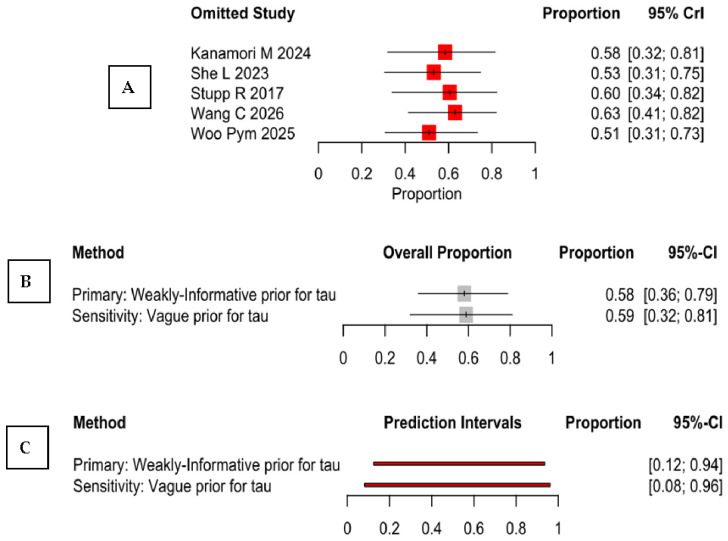
Robustness and prior-sensitivity analyses for dermatologic adverse effects. (**A**) LOO sensitivity analysis showing stable pooled proportions after sequential exclusion of individual studies [8,28,29,31,35]. (**B**) Summary of posterior adverse-effect proportions under different priors for between-study heterogeneity (τ). (**C**) Summary of posterior predictive distributions under different heterogeneity priors, illustrating the influence of τ prior choice on predictive uncertainty.

LOO sensitivity analysis confirmed the robustness of the primary findings for the outcome. Sequential omission of individual studies resulted in proportion values ranging from 0.51 to 0.63, with all 95% CrIs remaining below one. No single study exerted a disproportionate influence on the overall estimate (Figure 10A). Sensitivity analyses with different priors demonstrated that the pooled effect estimates were robust to prior specification, though credible intervals were expectedly wider when using less informative (vague) priors (Figure 10B). Furthermore, the posterior predictive distributions exhibited higher sensitivity to the choice of the between-study heterogeneity prior, with vague priors resulting in wider prediction intervals (Figure 10C).

### 3.3. Subgroup Analyses

#### 3.3.1. OS—Study Design

Regarding OS, in the RCT subgroup, the HR was 0.63 (95% CrI, 0.53; 0.75), and in the observational study subgroup, the HR was 0.68 (95% CrI, 0.58; 0.81). The overall (non-subgroup) posterior median HR was 0.68 (95% CrI 0.57; 0.79). There was a 63% PP that treatment effects differed between RCTs and observational studies. The residual between-study heterogeneity (τ), not explained by the subgroup covariate, was 0.19 (95% CrI 0.08; 0.34) (Figures S19 and S20).

Sensitivity analyses using alternative prior specifications for subgroup effects yielded consistent results, with overlapping overall effect; however, the use of vague priors resulted in wider credible intervals for the RCT subgroup and likewise for the observational study subgroup. PPs of benefit did not meaningfully change across prior specifications (Figure S21).

#### 3.3.2. PFS—Study Design

Regarding PFS, in the RCT subgroup, the HR was 0.63 (95% CrI, 0.53; 0.75), and in the observational study subgroup, the HR was 0.68 (95% CrI, 0.58; 0.79). The overall (non-subgroup) posterior median HR was 0.67 (95% CrI 0.59; 0.77). There was a 64% PP that treatment effects differed between RCTs and observational studies. The residual between-study heterogeneity (τ), not explained by the subgroup covariate, was 0.16 (95% CrI 0.05; 0.3) (Figures S22 and S23).

Sensitivity analyses using alternative prior specifications for subgroup effects yielded consistent results, with overlapping overall effect; however, the use of vague priors resulted in similar credible intervals for the RCT subgroup and likewise for the observational study subgroup. PPs of benefit did not meaningfully change across prior specifications (Figure S24).

#### 3.3.3. Dermatologic Adverse Effects—Study Design

Regarding adverse effects, in the RCT subgroup, the proportion was 0.48 (95% CrI, 0.43; 0.53), and in the observational study subgroup, the proportion was 0.59 (95% CrI, 0.35; 0.79). The overall (non-subgroup) posterior median HR was 0.58 (95% CrI 0.36; 0.78). There was a 62% PP that adverse effects differed between RCTs and observational studies (Figures S25 and S26). The residual between-study heterogeneity (τ), not explained by the subgroup covariate, was 0.94 (95% CrI 0.35; 1.78).

Sensitivity analyses using alternative prior specifications for subgroup effects yielded consistent results, with overlapping overall effect; however, the use of vague priors resulted in wider credible intervals for both the RCT subgroup and the observational study subgroup. PPs of harm did not meaningfully change across prior specifications (Figure S27).

#### 3.3.4. OS—ROB

In terms of OS, in the moderate-risk study subgroup, the HR was 0.65 (95% CrI, 0.53; 0.79); in the some-concerns risk study subgroup, the HR was 0.63 (95% CrI, 0.53; 0.75); and in the serious-risk study subgroup, the HR was 0.82 (95% CrI, 0.57; 1.15). The overall (non-subgroup) posterior median HR was 0.68 (95% CrI 0.57; 0.79) (Figures S28 and S29). The PP of the difference between the serious and moderate subgroups was 89%, and that between some concerns and moderate was 55% (Figure S30). The residual between-study heterogeneity (τ), not explained by the subgroup covariate, was 0.18 (95% CrI 0.06; 0.33).

Sensitivity analyses using alternative prior specifications for subgroup effects yielded consistent results, with overlapping overall effect; however, the use of vague priors resulted in wider credible intervals for the serious-risk subgroup, similar credible intervals for the moderate-risk subgroup, and likewise for the some-concerns risk subgroup. PPs of benefit did not meaningfully change across prior specifications (Figure S31).

#### 3.3.5. PFS—ROB

In terms of PFS, in the moderate-risk study subgroup, the HR was 0.64 (95% CrI, 0.53; 0.75); in the some-concerns risk study subgroup, the HR was 0.63 (95% CrI, 0.52; 0.76); and in the serious-risk study subgroup, the HR was 0.79 (95% CrI, 0.59; 1.05). The overall (non-subgroup) posterior median HR was 0.67 (95% CrI 0.59; 0.77) (Figures S32 and S33). The PP of the difference between the serious and moderate subgroups was 91%, and that between some concerns and moderate was 54% (Figure S34). The residual between-study heterogeneity (τ), not explained by the subgroup covariate, was 0.15 (95% CrI 0.04; 0.3).

Sensitivity analyses using alternative prior specifications for subgroup effects yielded consistent results, with overlapping overall effect; however, the use of vague priors resulted in similar credible intervals for the serious-risk subgroup, similar credible intervals for the moderate-risk subgroup, and likewise for the some-concerns risk subgroup. PPs of benefit did not meaningfully change across prior specifications (Figure S35).

#### 3.3.6. Dermatologic Adverse Effects—ROB

In terms of adverse effects, in the serious ROB risk subgroup, the proportion was 0.56 (95% CrI, 0.49; 0.63); in the some-concerns risk subgroup, the proportion was 0.48 (95% CrI, 0.43; 0.53); and in the moderate-risk subgroup, the proportion was 0.60 (95% CrI, 0.36; 0.81). The overall (non-subgroup) posterior median proportion was 0.58 (95% CrI 0.36; 0.79) (Figures S36 and S37). The PP of the difference between the serious and moderate subgroups was 55%, and that between some concerns and moderate was 63% (Figure S38). The residual between-study heterogeneity (τ), not explained by the subgroup covariate, was 0.98 (95% CrI 0.42; 1.85).

Sensitivity analyses using alternative prior specifications for subgroup effects yielded consistent results, with overlapping overall effect, but with wider credible intervals. Posterior probabilities of harm did not meaningfully change across prior specifications (Figure S39).

### 3.4. Quality Assessment

**Figure 11:**
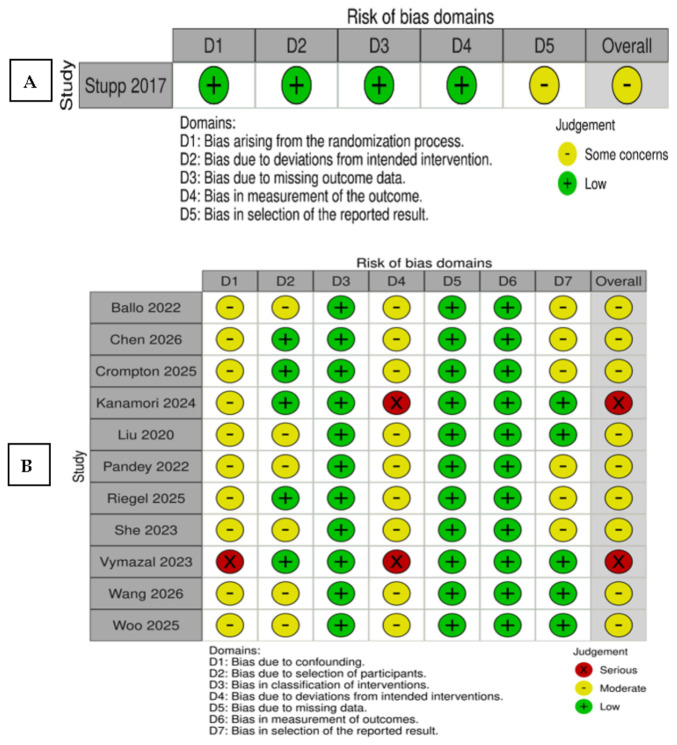
Risk of bias assessment of the included studies. (**A**) Risk-of-bias assessment of the included randomized controlled trial using the RoB 2.0 tool, showing some concerns, mainly related to selection of the reported result. (**B**) Risk-of-bias assessment of the included observational studies using ROBINS-I, showing moderate to serious risk of bias, with the main concerns related to confounding and deviations from intended interventions.

Among the twelve included studies, one was an RCT, and eleven were observational studies. Regarding the RCT, it was assessed as having some concerns of ROB according to the RoB 2.0 tool (Figure 11A). Among the observational studies, nine were rated as having a moderate ROB and two as having a serious ROB based on the ROBINS-I tool (Figure 11B).

In the RCT, the most common sources of bias were related to selection of the reported result (Domain 5), and in the observational studies, the most common sources of bias were related to confounding (Domain 1) and deviations from intended interventions (Domain 4).

**Figure 12:**
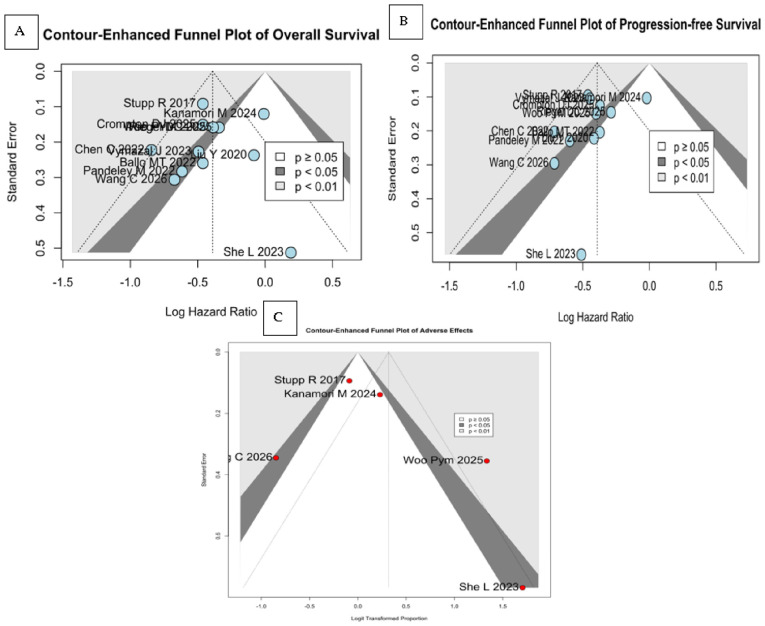
Funnel plots for publication bias assessment. (**A**) Funnel plot for overall survival (OS), showing no clear visual asymmetry; Egger’s test did not indicate publication bias (*p* = 0.6836). (**B**) Funnel plot for progression-free survival (PFS), also showing no clear asymmetry, with no evidence of publication bias by Egger’s test (*p* = 0.1954). (**C**) Funnel plot for dermatologic adverse effects, showing possible asymmetry; however, interpretation was limited by the small number of included studies.

Visual inspection of the funnel plots for OS and PFS did not suggest asymmetry, and Egger’s test provided no evidence of publication bias for either outcome (OS: *p* = 0.6836; PFS: *p* = 0.1954) (Figure 12A,B). By contrast, the funnel plot for dermatologic adverse effects appeared asymmetric, raising concern for possible publication bias, although visual interpretation was constrained by the small number of included studies (Figure 12C).

## 4. Discussion

For clinical readers, the Bayesian estimates can be interpreted directly as probabilities. For example, a posterior probability of 99% for HR < 1 means that, given the included studies and the specified model, there is a 99% probability that TTFs + TMZ is associated with better survival than TMZ alone. The credible interval represents the range of treatment effects most compatible with the observed data and prior assumptions. In contrast, the posterior predictive interval is broader because it asks a different question: what range of effects might be expected in a comparable future study after accounting for between-study heterogeneity. Thus, the predictive interval should be viewed as an exploratory measure of generalizability, not as a guarantee of future trial outcomes.

In this systematic review and Bayesian meta-analysis of 12 studies and 2797 patients, the addition of TTFs to TMZ was associated with a consistent survival benefit in nGBM, with improvements in both OS and PFS and very high PPs favoring TTFs + TMZ. Importantly, the predictive component of the Bayesian analysis extends the interpretation beyond statistical significance alone: the PPPs suggested that future exchangeable studies are also highly likely to demonstrate benefit. This is clinically relevant in nGBM, where therapeutic progress has historically been incremental and long-term survival remains uncommon despite the Stupp protocol [2,4,5]. Our findings therefore support TTFs as one of the few adjunctive strategies to show reproducible survival gains in this disease setting [8,9,10,28,29,30,31,32,33,34,35,36,37,38].

The potential benefit of combining TTFs with TMZ may be biologically plausible because the two modalities act through complementary mechanisms. TMZ induces DNA damage through alkylation, leading to tumor-cell apoptosis, particularly in tumors with impaired DNA repair capacity. In contrast, TTFs exert antimitotic effects by disrupting mitotic spindle formation, chromosome segregation, and cytokinesis. Experimental evidence also suggests that TTFs may interfere with DNA damage repair and increase cellular vulnerability to cytotoxic stress. Therefore, the combination of TMZ-related DNA injury and TTF-mediated mitotic disruption may produce additive or synergistic antitumor activity. This mechanistic rationale supports the clinical observation that TTFs + TMZ may improve survival compared with TMZ alone [8,9,10].

The magnitude of benefit observed here is broadly concordant with the randomized EF-14 trial and with more recent real-world studies from North America, Europe, and Asia included in our analysis [8,28,29,30,31,32,33,34,35,36,37,38]. This concordance is important because TTFs have sometimes been criticized on the basis of the open-label design of the pivotal trial, potential selection effects, and the practical dependence of efficacy on device adherence. However, the fact that our pooled estimates remained favorable after integrating both randomized and observational evidence, and that LOO analyses did not identify a single dominant study, argues against the survival signal being an isolated trial artifact. Moreover, the relatively narrow credible intervals for both OS and PFS strengthen the inference that the observed effect is not only statistically probable but also clinically meaningful [8,10,28,29,30,31,32,33,34,35,36,37,38].

A notable strength of the present study is the use of Bayesian methods. In contrast to conventional frequentist meta-analysis, the Bayesian framework allows direct probability statements about treatment benefit and expected future effects, which are often more interpretable for clinicians [13,14,20,21,22,23,24,39]. In our study, the PPs that HRs were below 1.0 were 99% for both OS and PFS, while the PPPs that future studies would also favor TTFs + TMZ were 96% and 98%, respectively. These results are especially valuable in a field such as neuro-oncology, where evidence synthesis frequently involves small numbers of studies, mixed designs, and clinically heterogeneous populations. The robustness of the results across alternative prior specifications further supports the stability of the conclusions and reduces concern that the findings were materially driven by prior assumptions alone [13,14,20,21,22,23,24,25].

Our findings should also be interpreted in the context of prior frequentist evidence reviews. The 2023 meta-analysis by Ballo et al. supported a survival benefit with TTFs, but its review framework was broader than ours, incorporating both comparative and single-cohort studies and evaluating TTFs added to the standard of care rather than isolating the specific post-chemoradiation comparison of maintenance TTFs plus TMZ versus maintenance TMZ alone [10]. More recently, a 2026 frequentist meta-analysis reported pooled hazard ratios for OS and PFS that were numerically similar to ours, which is reassuring regarding the direction and consistency of effect [40]. However, our Bayesian synthesis was intentionally more clinically focused and inferentially distinct: we applied a narrower PICOTT framework limited to the adjuvant post-Stupp setting, excluded non-comparative designs and clinically mixed populations without separately extractable GBM-specific data, and quantified not only pooled effects but also posterior probabilities and posterior predictive probabilities for future studies. We believe this design improves interpretability because it targets the incremental value of adding TTFs specifically to maintenance TMZ, reduces ambiguity arising from heterogeneous comparator definitions, and minimizes the possibility that single-cohort or mixed-population evidence could obscure the causal contrast of interest. In this sense, our results do not merely replicate prior frequentist findings; they refine them by focusing on the most clinically relevant treatment decision and by providing probabilistic estimates that may be more directly applicable to clinical decision-making.

However, our meta-analysis identified a moderate degree of between-study heterogeneity (τ = 0.18; 95% CrI: 0.08–0.32), indicating variability in effect estimates across studies. This heterogeneity is consistent with the inconsistencies reported in previously published studies and underscores the influence of differing study designs, populations, and methodological approaches. For instance, a large Japanese multicenter cohort of 537 patients found that TTField use was not a statistically significant prognostic factor for OS, despite achieving compliance rates comparable to the EF-14 trial [35]. Similarly, a retrospective analysis of 104 patients by Liu et al. reported an increased 6-month (*p* = 0.006) and 1-year (*p* = 0.170) survival rate but failed to observe any long-term improvement in OS using a Cox proportional hazards model, suggesting the early benefits might have been partially driven by selection bias [34]. Furthermore, a single-center study in a Chinese cohort noted an absolute improvement in median OS (24.8 months vs. 18.6 months) that did not reach statistical significance (*p* = 0.368), likely due to sample size limitations [31]. Our Bayesian predictive distribution elegantly models these variations, predicting a 95% probability that the true HR of a future study will lie between 0.43 and 1.05. This interval accurately reflects the reality that while the vast majority of future cohorts will experience a survival benefit (96% PPP of HR < 1), occasional cohorts may show an HR approaching or slightly exceeding 1.0 due to unmeasured confounding variables.

Our subgroup analyses also provide useful insight into heterogeneity. Differences by study design appeared modest, whereas risk of bias explained heterogeneity more convincingly, with studies at serious risk of bias tending to show attenuated effects. This pattern is methodologically reassuring. If anything, it suggests that the survival advantage of TTFs + TMZ is not inflated by less rigorous studies and may be more conservative when sources of confounding are considered explicitly. At the same time, the persistence of favorable point estimates in both randomized and observational strata supports the external validity of the treatment effect across routine practice settings [8,28,29,30,31,32,33,34,35,36,37,38]. Because most included studies were observational, the findings should not be interpreted as equivalent to evidence from multiple randomized trials. Nevertheless, several features support the robustness of the conclusion: the survival benefit was directionally consistent across most included studies, leave-one-out analyses did not identify a single dominant study, sensitivity analyses using alternative priors yielded similar estimates, and subgroup analyses by risk of bias showed that the beneficial association persisted in studies with moderate risk of bias. However, the more attenuated estimates observed in serious-risk studies indicate that residual confounding and selection bias remain important considerations. Therefore, our conclusions are framed as evidence of a highly probable association between TTFs + TMZ and improved survival, rather than definitive proof of a causal effect across all clinical settings.

Our meta-analysis’s between-study heterogeneity can also be explained by variations in the molecular biology and surgical management of the included cohorts. TTFields’ interaction with the tumor genome is an evolving area of study. Pandey et al. conducted a deep next-generation sequencing analysis of 112 patients to identify specific genomic factors affecting TTField response. They discovered that molecular driver alterations in NF1, alongside wild-type PIK3CA and wild-type EGFR, were significantly associated with increased benefit from TTFields. When these three factors were combined into a “Molecular Survival Score”, they strongly correlated with improved OS and PFS. Notably, tumor TP53 status did not impact TTField efficacy [33]. Furthermore, standard prognostic markers like MGMT promoter methylation remain independent favorable prognosticators alongside TTField therapy [28,29,38].

The biological plausibility of our findings is strong. TTFs exert antitumor activity through multiple complementary mechanisms, including disruption of mitotic spindle assembly, abnormal chromosome segregation, cytokinetic failure, and broader downstream effects on DNA damage responses and tumor cell viability [7,39,41]. Preclinical and translational data also suggest that TTFs may enhance immunogenic cell death and interact favorably with other anticancer modalities, providing a rationale for their additive effect alongside TMZ [39,41]. Thus, the survival benefit observed in this meta-analysis is mechanistically credible rather than merely empiric.

From a practical perspective, adherence is likely central to maximizing benefit. Prior analyses of EF-14 and real-world cohorts have shown that higher compliance, often operationalized as use for at least 75% of the day, is associated with superior outcomes [42,43]. This observation may explain some of the between-study variation in effect size and has direct implications for clinical practice: the efficacy of TTFs should not be viewed only as a property of the device itself but also as a function of sustained treatment exposure, patient counseling, caregiver support, and proactive toxicity management [11,12,42,43]. In this context, our results may best reflect the effectiveness of a treatment strategy that includes both device therapy and the infrastructure needed to maintain adherence.

The degree of upfront surgical debulking is heavily tied to TTField outcomes. Ballo et al. and Wang et al. both reported that the EOR was significantly associated with OS in multivariate analyses (*p* = 0.02 and *p* = 0.032, respectively) [29,38]. Interestingly, She et al. noted that patients specifically with STR who used TTFields had a remarkably better PFS than those who did not (*p* = 0.003), suggesting that TTFields may play a critical role in controlling bulky residual disease [31]. The anomaly of the Kanamori et al. study, which found no PFS or OS benefit for TTFields in 537 Japanese patients despite good compliance, requires explanation [35]. This failure to replicate EF-14 outcomes may be driven by genomic differences between Asian and Caucasian populations, as noted in the literature regarding population-level genetic variations [44,45]. It is theorized that different baseline rates of EGFR gene amplification in Asian populations may render the tumors inherently less susceptible to the standard 200 kHz frequency of TTFields [35]. However, this resistance is not uniform across all Asian populations, as multiple Chinese cohorts have demonstrated massive survival benefits [28,31,37,38].

An emerging phenomenon associated with TTFields is the alteration of the natural history of GBM recurrence. Because TTFields are a locoregional therapy applied directly to the shaved scalp via arrays, the electric field intensity is highest in the cerebral cortex directly beneath the arrays and within the primary tumor bed. Crompton et al. mapped the failures of TTField patients and found a trend toward decreased in-field failures and increased marginal failures with the addition of TTFields (*p* = 0.099) [36]. Riegel et al. explicitly confirmed this, demonstrating that patients treated with TTFields exhibited a statistically higher rate of non-local (distant or marginal) progression compared to the non-TTField group [32]. This suggests that TTFields are highly effective at suppressing mitosis within the targeted radiation/resection field. However, motile GBM cells that escape the physical boundaries of the highest-intensity electric fields eventually establish distant recurrences. This shifting pattern of failure validates the profound local antimitotic efficacy of the device while highlighting the need for systemic therapies to address distant micrometastatic spread within the central nervous system.

Dermatologic toxicity was the principal trade-off identified in this study. The pooled estimated proportion was high, and predictive intervals suggested that future studies will often report clinically relevant rates. Nevertheless, available evidence indicates that these events are usually localized, low-grade, and manageable with scalp care, array optimization, topical therapies, and temporary treatment adjustments rather than discontinuation [11,12,46]. Importantly, randomized and real-world quality-of-life analyses suggest that TTFs do not appear to cause major deterioration in global health-related quality of life, cognitive function, or functional status, apart from increased scalp irritation and treatment burden related to device wear [39,46,47]. These considerations are important when counseling patients, because the balance between survival prolongation and day-to-day tolerability is especially consequential in glioblastoma care. From a clinical perspective, the benefit of TTFs + TMZ should be interpreted as a balance between survival prolongation, treatment burden, device adherence, quality of life, and toxicity. Dermatologic adverse effects such as erythema, pruritus, contact dermatitis, erosions, ulcerations, and discomfort are common because transducer arrays are continuously applied to the scalp; these toxicities are clinically relevant because they may reduce adherence to daily device use; however, these events are usually localized and can often be managed with preventive scalp care, repositioning of arrays, topical corticosteroids or antibiotics when appropriate, temporary interruption of TTFs, patient education, and close dermatologic monitoring. Therefore, patient benefit is greatest when individuals have adequate performance status, are motivated to maintain device use, have sufficient caregiver or institutional support, and can receive early management of skin toxicity. Shared decision-making is essential, because some patients may prioritize survival extension, whereas others may place greater weight on daily device burden, cost, cosmetic concerns, or quality-of-life implications.

Our findings also have implications for patient selection and health-system adoption. Contemporary consensus guidance recognizes TTFs as a treatment option for selected patients with nGBM, but uptake remains variable across centers and jurisdictions [48,49]. This variability likely reflects not only clinical uncertainty but also logistical, socioeconomic, and reimbursement barriers. Cost-effectiveness analyses have produced mixed conclusions across settings, suggesting that the value proposition of TTFs depends strongly on local pricing structures, willingness-to-pay thresholds, and healthcare-system context [50,51]. Therefore, while our results support the efficacy of TTFs + TMZ, implementation should still be individualized and accompanied by shared decision-making [48,49,50,51].

This study has several limitations. First, only one RCT was available, while most included studies were observational. Therefore, residual confounding, treatment-selection bias, and differences in supportive care cannot be excluded, even though subgroup analyses according to study design and risk of bias were performed. Second, patient-level effect modifiers could not be uniformly synthesized. Important variables such as MGMT promoter methylation, IDH status, extent of resection, corticosteroid exposure, performance status, tumor location, socioeconomic factors, and actual TTF adherence were inconsistently reported across studies. Third, the adverse-event analysis was based on fewer studies than the survival analyses and showed substantial between-study heterogeneity, likely reflecting differences in adverse-event definitions, reporting practices, follow-up duration, and dermatologic management. Fourth, although Bayesian posterior predictive analyses improve clinical interpretability by incorporating between-study heterogeneity, they should not be interpreted as definitive forecasts of future trial results. They represent model-based estimates under the assumption that future populations are exchangeable with the included studies. Fifth, the restriction to English-language articles and exclusion of the gray literature may have introduced language and publication bias. Finally, cost, reimbursement, device availability, and patient adherence were not uniformly available for quantitative synthesis, although these factors are highly relevant to real-world implementation.

Overall, this meta-analysis indicates that TTFs + TMZ provides a robust and highly probable survival advantage over TMZ alone in nGBM, with dermatologic toxicity that is frequent but generally manageable. Future research should prioritize prospective real-world cohorts and individual-patient-data analyses to refine patient selection, quantify the role of adherence, and clarify whether molecular subgroups derive differential benefit from TTF-based therapy.

## 5. Conclusions

In this Bayesian meta-analysis, the addition of TTFs to TMZ was associated with a robust and clinically meaningful improvement in both OS and PFS in patients with nGBM, with high posterior and predictive probabilities supporting the consistency of this benefit. Although dermatologic adverse effects were frequent, they were largely localized and should be weighed against the survival advantage observed with this multimodal approach. Taken together, these findings support TTFs plus TMZ as an effective adjunctive strategy for nGBM and highlight the importance of optimizing adherence, toxicity management, and patient selection in future prospective and real-world studies.

## Figures and Tables

**Table 1:** Baseline characteristics of the included studies.

Study	Country/Center	Design	Level of Evidence	No. of Patients	TTFs + TMZ	TMZ	Age *	Female, %	Performance Status (KPS/ECOG)	Type of Surgery	MGMT Promoter	IDH Status	Tumor Location	Follow-Up, Months
Ballo, 2022 [38]	USA, single-center	Retrospective cohort	IIb	91	59	32	TTFs + TMZ: 59 TMZ: 63	TTFs + TMZ: 32.2 TMZ: 31.3	ECOG 0–1: 80 (87.9%)	GTR 60.4%; STR 27.5%; biopsy 12.1%	Methylated: 43 (47.3%)	IDH wild-type (100%)	Frontal 29 (31.9%); Parietal 22 (24.2%); Temporal 32 (35.2%)	26.5
Chen, 2022 [37]	China, single-center	Retrospective cohort	IIb	267	63	204	TTFs + TMZ: 51 TMZ: 54	TTFs + TMZ: 52.0 TMZ: 35.3	KPS: TTFs + TMZ 80; TMZ 90	GTR 76.8%; STR 19.9%; biopsy 3.4%	MGMT methylated: TTFs + TMZ 32%; TMZ 21%	IDH wild-type: TTFs + TMZ 89%; TMZ 88%	Frontal 29% vs. 42%; Superficial 41% vs. 47%; Deep 30% vs. 11%	18.4
Crompton, 2025 [36]	USA, multicenter	Retrospective cohort	IIb	393	74	319	TTFs + TMZ: 57.6 TMZ: 61.5	TTFs + TMZ: 40.5 TMZ: 37.3	ECOG: TTFs + TMZ 0–1 (87%); TMZ 0–1 (86%)	GTR 51.1%; STR 27.5%; biopsy 21.4%	MGMT methylated: TTFs + TMZ 43.2%; TMZ 43.3%	IDH wild-type: TTFs + TMZ 91.9%; TMZ 94.4%	N/A	26.4
Kanamori, 2024 [35]	Japan, multicenter	Retrospective cohort	IIb	537	210	327	TTFs + TMZ: 54.0 TMZ: 60.0	TTFs + TMZ: 37 TMZ: 43	KPS: TTFs + TMZ ≥ 90 (61%); TMZ ≥ 90 (52%)	GTR 64.2%; STR 28.7%; biopsy 7.1%	MGMT methylated: TTFs + TMZ 21%; TMZ 21%	IDH wild-type: TTFs + TMZ 92%; TMZ 95%	Frontal 39% vs. 34%; Temporal 28% vs. 32%; Parietal 24% vs. 25%; Occipital 2% vs. 4%;	21
Liu, 2020 [34]	USA, single-center	Retrospective cohort	IIb	104	37	67	TTFs + TMZ: 61 TMZ: 65	TTFs + TMZ: 37.8 TMZ: 43.3	KPS: TTFs + TMZ > 80 (78.4%); TMZ > 80 (59.7%)	GTR 50.0%; STR 36.5%; biopsy 13.5%	MGMT methylated: TTFs + TMZ 16.2%; TMZ 35.8%	IDH wild-type: TTFs + TMZ 89.2%; TMZ 82.1%	N/A	42
Pandey, 2022 [33]	USA, multicenter	Retrospective cohort	IIb	112	55	57	TTFs + TMZ: 59 TMZ: 58	TTFs + TMZ: 31 TMZ: 40	N/A	N/A	MGMT methylated: TTFs + TMZ 45%; TMZ 46%	IDH mutation: TTFs + TMZ 9%; TMZ 5%	Temporal 29% vs. 18%; Frontal 25% vs. 30%; Parietal 16% vs. 21%;	~24
Riegel, 2025 [32]	USA, single-center	Retrospective cohort	IIb	208	109	99	TTFs + TMZ: 60 TMZ: 64	TTFs + TMZ: 43 TMZ: 43	N/A	GTR 38.5%; STR 52.9%; biopsy 8.7%	MGMT methylated: TTFs + TMZ 34%; TMZ 31%	IDH wild-type: TTFs + TMZ 94%; TMZ 93%	Frontal 32% vs. 32%; Temporal 24% vs. 31%; Parietal 18% vs. 20%;	21.7
She, 2023 [31]	China, single-center	Retrospective cohort	IIb	52	13	39	TTFs + TMZ: 54 TMZ: 48	TTFs + TMZ: 46.2 TMZ: 38.5	KPS: TTFs + TMZ > 70 (69.2%); TMZ > 70 (76.9%)	GTR 55.8%; STR 44.2%; biopsy 0%	MGMT methylated: TTFs + TMZ 23.1%; TMZ 33.3%	IDH wild-type (100% both groups)	Frontal/temporal/parietal/occipital: 61.5% vs. 89.7%; Corpus callosum: 30.8%	34.7
Stupp, 2017 [8]	Multinational	RCT	Ib	695	466	229	TTFs + TMZ: 56 TMZ: 57	TTFs + TMZ: 32 TMZ: 31	KPS: TTFs + TMZ 90; TMZ 90	GTR 53.5%; STR 33.7%; biopsy 12.8%	MGMT methylated: TTFs + TMZ 36%; TMZ 42%	IDH mutation: TTFs + TMZ 7%; TMZ 5%	Frontal 41% vs. 37%; Temporal 41% vs. 40%; Parietal 31% vs. 39%; Occipital 12% vs. 12%	40
Wang, 2026 [29]	China, single-center	Retrospective cohort	IIb	88	40	48	TTFs + TMZ: 56.4 TMZ: 57.3	TTFs + TMZ: 47.5 TMZ: 50.0	KPS > 60 (100% both groups)	GTR 81.8%; STR/biopsy 18.2%	MGMT methylated: TTFs + TMZ 45.0%; TMZ 39.6%	IDH wild-type (100% both groups)	N/A	11
Woo, 2025 [28]	Hong Kong, multicenter	Prospective observational	IIb	141	47	94	TTFs + TMZ: 54 TMZ: 53	TTFs + TMZ: 40 TMZ: 29	KPS ≥ 80: TTFs + TMZ 72%; TMZ 73%	GTR 100%; STR 0%; biopsy 0%	MGMT methylated: TTFs + TMZ 43%; TMZ 45%	IDH mutation: TTFs + TMZ 11%; TMZ 9%	Frontal 36% vs. 32%; Temporal 36% vs. 39%; Parietal 15% vs. 13%;	26.5
Vymazal, 2023 [30]	Czech Republic, single-center	Retrospective cohort	IIb	109	55	54	TTFs + TMZ: 47.3 TMZ: 51.4	TTFs + TMZ: 36 TMZ: 37	KPS: TTFs + TMZ 80; TMZ 80	GTR 74.3%; STR 24.8%; biopsy 0.9%	MGMT methylated: TTFs + TMZ 27.3%; TMZ 16.7%	IDH mutation: TTFs + TMZ 7.3%; TMZ 1.9%	Frontal 22% vs. 33%; Temporal 31% vs. 26%; Parietal 20% vs. 13%;	23.7

* Mean; ECOG—Eastern Cooperative Oncology Group performance status; GTR—gross total resection; IDH—isocitrate dehydrogenase; KPS—Karnofsky performance status; MGMT—O6-methylguanine-DNA methyltransferase; ndGBM—newly diagnosed glioblastoma; STR—subtotal resection; TMZ—temozolomide; TTFs—tumor-treating fields.

## Data Availability

All data are publicly available from the included studies.

## Supplementary Materials

The following supporting information can be downloaded at: https://www.mdpi.com/article/10.3390/cancers18121947/s1, Figure S1: Vague Normal (0, 2.7) prior for the overall effect (μ), with 95% of the probability density spanning a hazard ratio of 0.01 to 198.72 to represent a lack of prior knowledge.; Figure S2: Informative Log-Normal (−1.46, 0.51) prior for between-study heterogeneity, based on the predictive distribution for non-pharmacological comparisons (Turner et al., 2015); Figure S3: Weakly informative Half-Normal (0.52) prior for heterogeneity. The 95th percentile (0.98) corresponds to a plausible range of study effects between 0.15 and 6.82 on the hazard ratio scale; Figure S4: Vague Half-Normal (1.52) prior for heterogeneity. The 95th percentile (2.94) allows for extreme study effects ranging from 0.003 to 318 on the hazard ratio scale, representing a non-informative approach; Figure S5: Vague Normal (0, 2.7) prior for the overall effect (μ), with 95% of the probability density spanning a hazard ratio of 0.01 to 198.72 to represent a lack of prior knowledge; Figure S6: Informative Log-Normal (−1.34, 0.755) prior for between-study heterogeneity, based on the predictive distribution for non-pharmacological comparisons (Turner et al., 2015); Figure S7: Weakly informative Half-Normal (0.52) prior for heterogeneity. The 95th percentile (0.98) corresponds to a plausible range of study effects between 0.15 and 6.82 on the hazard ratio scale; Figure S8: Vague Half-Normal (1.52) prior for heterogeneity. The 95th percentile (2.94) allows for extreme study effects ranging from 0.003 to 318 on the hazard ratio scale, representing a non-informative approach; Figure S9: Weakly informative prior for overall effect; Figure S10: Weakly informative prior Half-Normal (1) for the heterogeneity; Figure S11: Vague prior Half-Normal (2) for heterogeneity; Figure S12: Vague prior distribution for the reference group overall effect, using Normal (0, 2.72) for binary outcomes (log scale); Figure S13: Weakly informative Normal (0, 0.82) prior for subgroup differences on the log scale. The distribution assumes large differences are unlikely, with the 95% density for the Ratio of Hazard Ratios (RHR) ranging from 0.20 to 5.00; Figure S14: Weakly informative Normal (0, 0.71) prior for subgroup differences on the log scale. The distribution assumes large differences are unlikely, with the 95% density for the proportions ranging from 0.25 to 4.02; Figure S15: Sensitivity analysis prior for heterogeneity (τ): a weakly informative Half-Normal (0.5) distribution used to assess the robust-ness of subgroup findings; Figure S16: Sensitivity analysis prior for heterogeneity (τ): a weakly informative Half-Normal (1.5) distribution used to assess the robustness of subgroup findings; Figure S17: Sensitivity analysis prior for heterogeneity (τ) in proportions: a weakly informative Half-Normal (1) distribution used to assess the robustness of subgroup findings; Figure S18: Sensitivity analysis prior for heterogeneity (τ) in proportions: a vague Half-Normal (2) distribution used to assess the robustness of subgroup findings; Figure S19: Forest plot showing subgroup analysis of OS by study design. The pooled Hazard Ratio was 0.68 (95% CrI, 0.58–0.81) for observational studies and 0.63 (95% CrI, 0.53–0.75) for RCTs, with a 64% posterior probability of a subgroup difference; Figure S20: Posterior distributions of subgroup-specific treatment effects. Left panel shows posterior densities for observational studies, RCTs, and overall pooled effect (hazard ratio, log scale). Right panel depicts posterior probabilities that the hazard ratio exceeds 1 across subgroups; Figure S21: Sensitivity analysis for the observational and RCT subgroup under alternative prior specifications. Forest plot showing posterior hazard ratios and 95% CrI using informative, weakly informative, and vague priors for heterogeneity. Estimates remained directionally consistent across prior assumptions; Figure S22: Forest plot showing subgroup analysis of PFS by study design. The pooled Hazard Ratio was 0.68 (95% CrI, 0.58–0.79) for observational studies and 0.63 (95% CrI, 0.53–0.75) for RCTs, with a 64% posterior probability of a subgroup difference; Figure S23: Posterior distributions of subgroup-specific treatment effects. Left panel shows posterior densities for observational studies, RCTs, and overall pooled effect (hazard ratio, log scale). Right panel depicts posterior probabilities that the hazard ratio exceeds 1 across subgroups; Figure S24: Sensitivity analysis for the observational and RCT subgroup under alternative prior specifications. Forest plot showing posterior hazard ratios and 95% CrI using informative, weakly informative, and vague priors for heterogeneity. Estimates remained directionally consistent across prior assumptions; Figure S25: Forest plot showing subgroup analysis of dermatological adverse effects by study design. The pooled proportions were 0.59 (95% CrI, 0.35–0.79) for observational studies and 0.48 (95% CrI, 0.43–0.53) for RCTs, with a 62% posterior probability of a subgroup difference; Figure S26: Posterior distributions of subgroup-specific treatment effects. Left panel shows posterior densities for observational studies, RCTs, and overall pooled effect (proportions). Right panel depicts posterior probabilities for harm across subgroups; Figure S27: Sensitivity analysis for the observational and RCT subgroup under alternative prior specifications. Forest plot showing posterior proportions and 95% CrI using weakly informative, and vague priors for heterogeneity. Estimates remained directionally consistent across prior assumptions; Figure S28: Forest plot showing subgroup analysis of OS by risk of bias. The pooled Hazard Ratio was 0.65 (95% CrI, 0.53–0.79) for moderate risk studies, 0.63 (95% CrI, 0.53–0.75) in some concerns risk studies, and 0.82 (95% CrI, 0.57–1.15) for serious risk; Figure S29: Posterior distributions of subgroup-specific treatment effects. Left panel shows posterior densities for moderate risk, some concerns, serious risk studies, and overall pooled effect (hazard ratios). Right panel depicts posterior probabilities for benefit across subgroups; Figure S30: Posterior density plots for pairwise subgroup comparisons. The overall posterior probability of a difference between some concerns and moderate risk studies was 55%, and between serious and moderate risk studies—89% respectively; Figure S31: Sensitivity analysis for the some concerns, moderate, and serious risk subgroups under alternative prior specifications. Forest plot showing posterior hazard ratios and 95% CrI using informative, weakly informative, and vague priors for heterogeneity. Estimates remained directionally consistent across prior assumptions; Figure S32: Forest plot showing subgroup analysis of PFS by risk of bias. The pooled Hazard Ratio was 0.64 (95% CrI, 0.53–0.75) for moderate risk studies, 0.63 (95% CrI, 0.52–0.76) in some concerns risk studies, and 0.79 (95% CrI, 0.59–1.05) for serious risk; Figure S33: Posterior distributions of subgroup-specific treatment effects. Left panel shows posterior densities for moderate risk, some concerns, serious risk studies, and overall pooled effect (hazard ratios). Right panel depicts posterior probabilities for benefit across subgroups; Figure S34: Posterior density plots for pairwise subgroup comparisons. The overall posterior probability of a difference between some concerns and moderate risk studies was 54%, and between serious and moderate risk studies—91% respectively; Figure S35: Sensitivity analysis for the some concerns, moderate, and serious risk subgroups under alternative prior specifications. Forest plot showing posterior hazard ratios and 95% CrI using informative, weakly informative, and vague priors for heterogeneity. Estimates remained directionally consistent across prior assumptions; Figure S36: Forest plot showing subgroup analysis of dermatologic adverse effects by risk of bias. The pooled proportion was 0.60 (95% CrI, 0.36–0.81) for moderate risk studies, 0.48 (95% CrI, 0.43–0.53) in some concerns risk studies, and 0.56 (95% CrI, 0.49–0.63) for serious risk; Figure S37: Posterior distributions of subgroup-specific treatment effects. Left panel shows posterior densities for moderate risk, some concerns, serious risk studies, and overall pooled effect (proportions). Right panel depicts posterior probabilities for harm across subgroups; Figure S38: Posterior density plots for pairwise subgroup comparisons. The overall posterior probability of a difference between some concerns and moderate risk studies was 63%, and between serious and moderate risk studies—55% respectively; Figure S39: Sensitivity analysis for the some concerns, moderate, and serious risk subgroups under alternative prior specifications. Forest plot showing posterior proportions and 95% CrI using weakly informative, and vague priors for heterogeneity. Estimates remained directionally consistent across prior assumptions.

## Abbreviations

Tumor-treating fields (TTFs), temozolomide (TMZ), newly diagnosed glioblastoma (nGBM), overall survival (OS), progression-free survival (PFS), hazard ratio (HR), credible interval (CrI), posterior probability (PP), posterior predictive probability (PPP), randomized controlled trial (RCT), risk of bias (ROB), Karnofsky performance status (KPS), Eastern Cooperative Oncology Group (ECOG), gross total resection (GTR), subtotal resection (STR), O6-methylguanine-DNA methyltransferase (MGMT), and isocitrate dehydrogenase (IDH).
