# Supplementary material for: Tumor-Treating Fields Plus Temozolomide Versus Temozolomide Alone in Newly Diagnosed Glioblastoma: A Systematic Review and Bayesian Meta-Analysis with Meta-Regression
Source: Cancers (Basel). 2026 Jun 16;18(12):1947. doi: 10.3390/cancers18121947 (PMC13297347; doi:10.3390/cancers18121947)
Supplement: Supplementary file 1 [file cancers-18-01947-s001.zip › cancers-4314063-supplementary.pdf]

Supplementary METHODS

Bayesian analysis

Bayesian meta-analyses were conducted using the R package bayesmeta [25]. All analyses were conducted in R (R Environment version 4.3.1) [26].

We fitted a random-effect meta-analysis model through the Bayesian framework, which updates prior beliefs with the current data to form a posterior distribution [20]. In the primary analysis, for the overall effect, we applied a vague prior to all analyses, and, for the between-study heterogeneity parameter, an informative prior was applied. We also fitted models with different priors in sensitivity analyses to check whether this choice of priors meaningfully impacted our results or our conclusions.

Bayesian random-effects meta-analysis yields marginal posterior distributions for the overall effect and between-study heterogeneity. We used mean and 95% highest-density intervals (hereafter, credible intervals [CrI]) to describe these distributions, defined as the narrowest interval containing 95% of the probability density function [21]. Our primary estimands are expressed as Hazard Ratios (HRs) and proportions (%) for binary outcomes, and we also focused on the calculation of posterior probabilities of clinically relevant effects.

For each outcome, we fitted a Bayesian random-effects meta-analysis model. This model consists of the observed mean log odds ratio and the mean difference from each study. We assume these effect sizes are normally distributed around the true study-specific means along with a known sampling variance. We also assume that these study-specific means are drawn from another normal distribution where "mu" is the average effect (mean) and "tau" is the between-study heterogeneity (standard deviation).

Between-study heterogeneity and prediction

By accounting for both within and between study variation, random-effects meta-analysis can better incorporate overall heterogeneity [20,22]. In the Bayesian framework, one acknowledges the prior uncertainty in the heterogeneity parameter and can estimate the posterior distribution of the between-study heterogeneity.

Further, the posterior predictive distribution can better explore the impact of between-study heterogeneity. This distribution allows inference about "…what we would expect to see in a new study population that is exchangeable with the studies included in our meta-analysis" [23]. The posterior predictive distribution is vital to inform probable values for the true treatment association in future settings. This distribution incorporates the uncertainty both in the overall effect size and the between-study standard deviation parameters and generates new trial population parameter estimates, independently of sample size and other population characteristics [24].

Overall Survival – Hazard Ratios (HR)

1. Primary analysis: Vague for "mu", Informative for "tau"
2. Sensitivity analysis 1: Vague for "mu", Weakly informative" for "tau"
3. Sensitivity analysis 2: Vague for "mu", Vague for "tau"

Because these are Bayesian models, we need to specify prior distributions for all parameters. In the primary analysis, we applied a vague prior for the overall effect ("mu"), where all relevant values are equally likely, corresponding to no prior knowledge for the

average effect: Normal(0, 2.7), 95% of the probability density ranging from 0.01 to 198.72 hazard ratio (log scale), as depicted below (Figure S1):

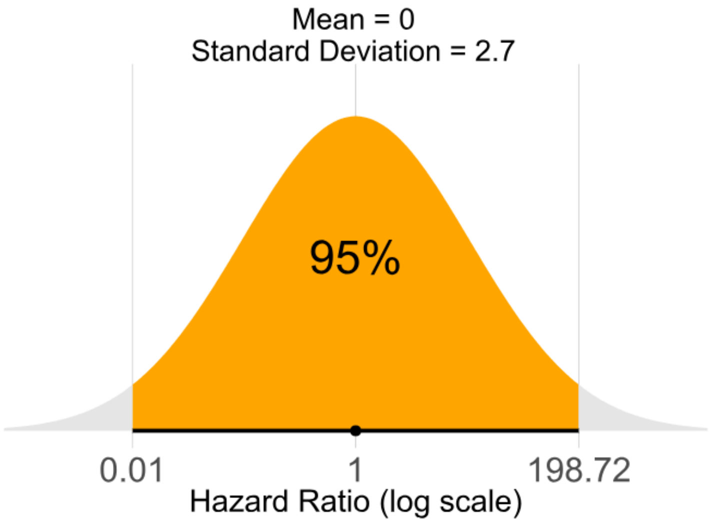

Figure S1. Vague Normal(0, 2.7) prior for the overall effect (μ), with 95% of the probability density spanning a hazard ratio of 0.01 to 198.72 to represent a lack of prior knowledge.

For the between-study heterogeneity parameter, we used an informative prior, Log-Normal (-1.46, 0.51), based on the predictive distribution derived from hundreds of Cochrane meta-analyses (Turner et al., 2015). We applied the predictive distribution on all-cause mortality in TurnerEtAl() for non-pharmacological in TurnerEtAl() vs. pharmacological in TurnerEtAl() comparisons derived by Turner et al (Figure S2).

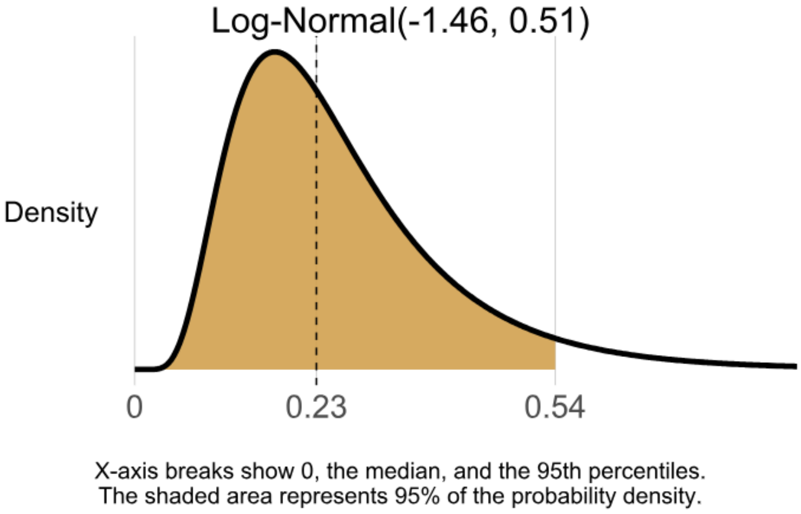

Figure S2. Informative Log-Normal(-1.46, 0.51) prior for between-study heterogeneity, based on the predictive distribution for non-pharmacological comparisons (Turner et al., 2015).

We also did two separate sensitivity analyses to test the robustness of our results to different priors. While we applied the same vague prior for "mu" in both sensitivity analyses, we tested different priors for "tau".

In the first analysis, we used a weakly informative prior, Half-Normal(0.52), recommended by Rover et al. 2021 [16] (Figure S3). The 95th percentile of this half-normal distribution is 0.98 – log scale. Considering a normal distribution with a mean of 0 and a

standard deviation of 0.98, 95% of true study effects would range from 0.15 to 6.82 hazard ratio – linear scale. We consider these values plausible, assigning limited density on extreme values, compatible with the concept of a "weakly informative" prior.

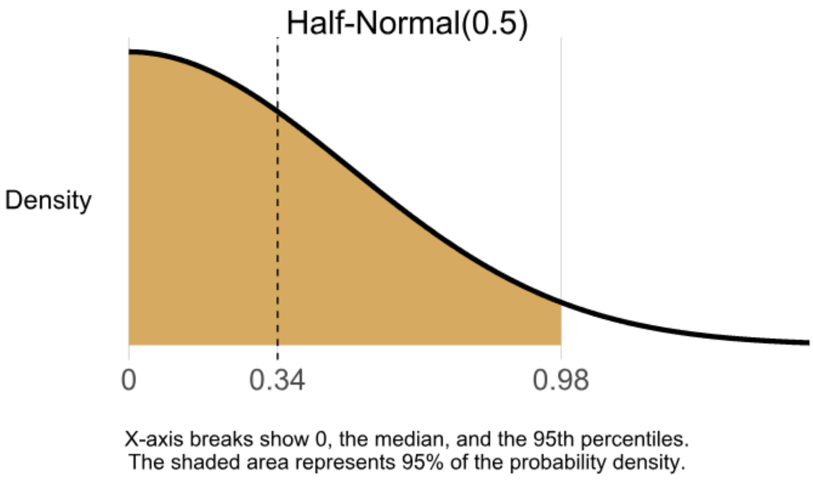

Figure S3. Weakly informative Half-Normal(0.52) prior for heterogeneity. The 95th percentile (0.98) corresponds to a plausible range of study effects between 0.15 and 6.82 on the hazard ratio scale.

In the second analysis, we used a vague prior, Half-Normal(1.52). The 95th percentile of this half-normal distribution is 2.94 – log scale (Figure S4). Considering a normal distribution with a mean of 0 and a standard deviation of 2.94, 95% of true study effects would range from 0.003 to 318 hazard ratio – linear scale. In this case, extreme values are as likely as plausible values, compatible with the concept of vague (or "flat"; or "non-informative") prior.

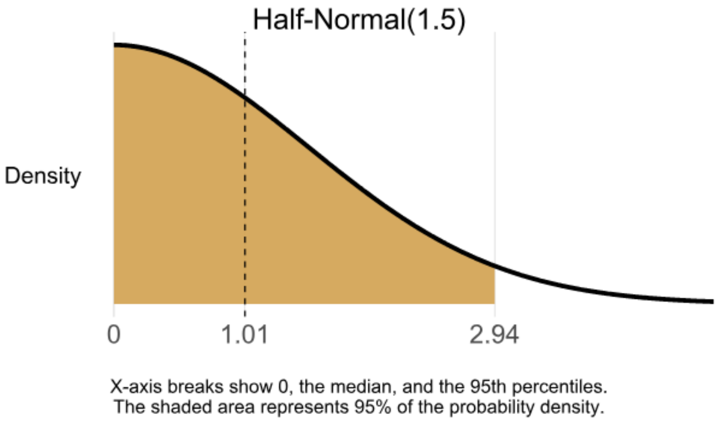

Figure S4. Vague Half-Normal(1.52) prior for heterogeneity. The 95th percentile (2.94) allows for extreme study effects ranging from 0.003 to 318 on the hazard ratio scale, representing a non-informative approach.

Progression-Free Survival – HR

- Primary analysis: Vague for "mu", Informative for "tau"
- Sensitivity analysis 1: Vague for "mu", Weakly informative" for "tau"
- Sensitivity analysis 2: Vague for "mu", Vague for "tau"

Because these are Bayesian models, we need to specify prior distributions for all parameters. In the primary analysis, we applied a vague prior for the overall effect ("mu"),

where all relevant values are equally likely, corresponding to no prior knowledge for the average effect: Normal(0, 2.7), 95% of the probability density ranging from 0.01 to 198.72 hazard ratio (log scale), as depicted below (Figure S5).

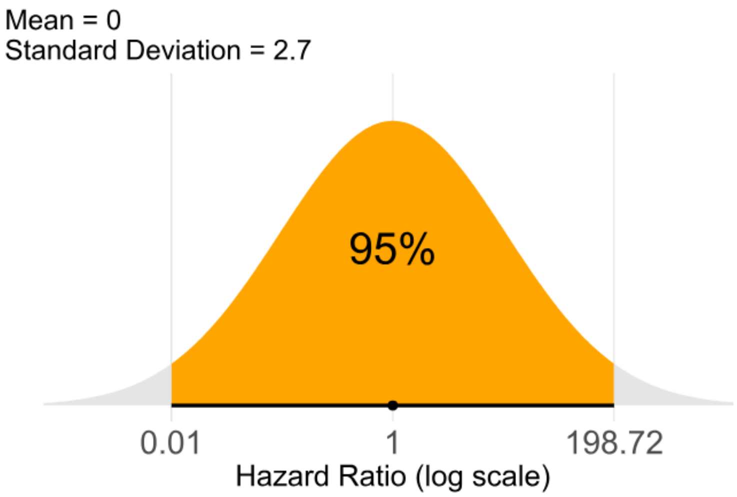

Figure S5. Vague Normal(0, 2.7) prior for the overall effect (μ), with 95% of the probability density spanning a hazard ratio of 0.01 to 198.72 to represent a lack of prior knowledge.

For the between-study heterogeneity parameter, we used an informative prior, Log-Normal (-1.46, 0.51), based on the predictive distribution derived from hundreds of Cochrane meta-analyses (Turner et al., 2015). We applied the predictive distribution on (cause-specific mortality / major morbidity event / composite (mortality or morbidity)) in TurnerEtAl() for non-pharmacological in TurnerEtAl() vs. pharmacological in TurnerEtAl() comparisons derived by Turner et al (Figure S6).

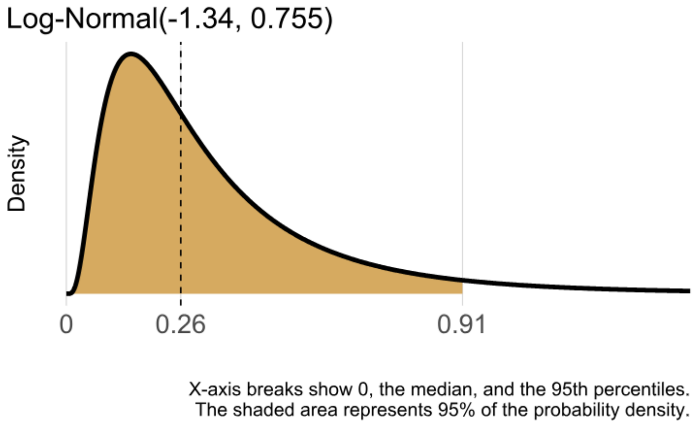

Figure S6. Informative Log-Normal(-1.34, 0.755) prior for between-study heterogeneity, based on the predictive distribution for non-pharmacological comparisons (Turner et al., 2015).

We also did two separate sensitivity analyses to test the robustness of our results to different priors. While we applied the same vague prior for "mu" in both sensitivity analyses, we tested different priors for "tau".

In the first analysis, we used a weakly informative prior, Half-Normal(0.52), recommended by Rover et al. 2021 [16] (Figure S7). The 95th percentile of this half-normal distribution is 0.98 – log scale. Considering a normal distribution with a mean of 0 and a standard deviation of 0.98, 95% of true study effects would range from 0.15 to 6.82 hazard

ratio – linear scale. We consider these values plausible, assigning limited density on extreme values, compatible with the concept of a "weakly informative" prior.

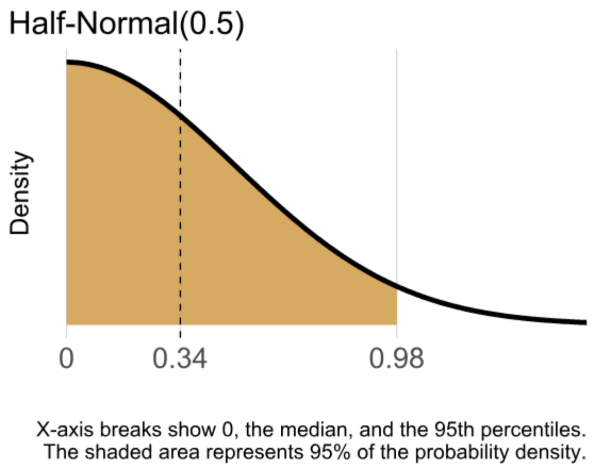

Figure S7. Weakly informative Half-Normal(0.52) prior for heterogeneity. The 95th percentile (0.98) corresponds to a plausible range of study effects between 0.15 and 6.82 on the hazard ratio scale.

In the second analysis, we used a vague prior, Half-Normal(1.52). The 95th percentile of this half-normal distribution is 2.94 – log scale (Figure S8). Considering a normal distribution with a mean of 0 and a standard deviation of 2.94, 95% of true study effects would range from 0.003 to 318 hazard ratio – linear scale. In this case, extreme values are as likely as plausible values, compatible with the concept of vague (or "flat"; or "non-informative") prior.

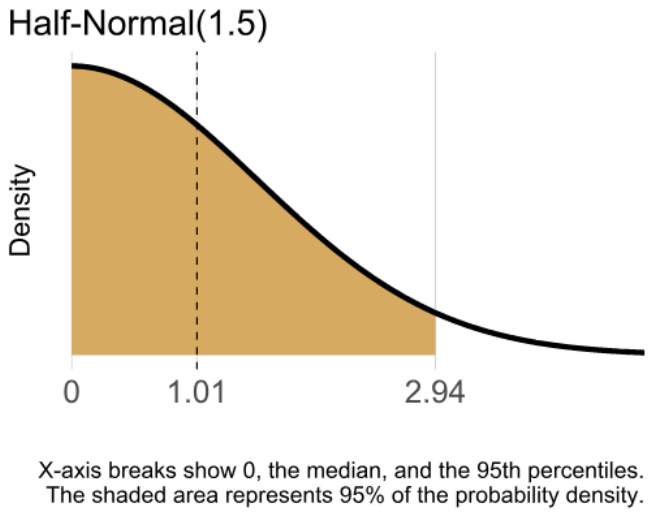

Figure S8. Vague Half-Normal(1.52) prior for heterogeneity. The 95th percentile (2.94) allows for extreme study effects ranging from 0.003 to 318 on the hazard ratio scale, representing a non-informative approach.

Dermatological Adverse Effects – Proportions

- Primary analysis: Weakly-informative for "mu", Weakly informative for "tau"
- Sensitivity analysis: Weakly informative for "mu", Vague for "tau"

Because these are Bayesian models, we need to specify prior distributions for all parameters. In the primary analysis, we applied a weakly informative prior for the overall log-odds ("mu"): Normal(0, 1.52) on the log-odds scale (Figure S9). Importantly, while

this prior appears wide on the log-odds scale, when transformed back to the proportion scale via the inverse-logit function (plogis()), it covers the full range of plausible proportions (approximately 5% to 95%) without overly concentrating density at extreme values near 0 or 1. Wider log-odds priors (e.g., Normal(0, 10^2)) would paradoxically favor proportions near 0 or 1, which is rarely appropriate for proportion meta-analyses:

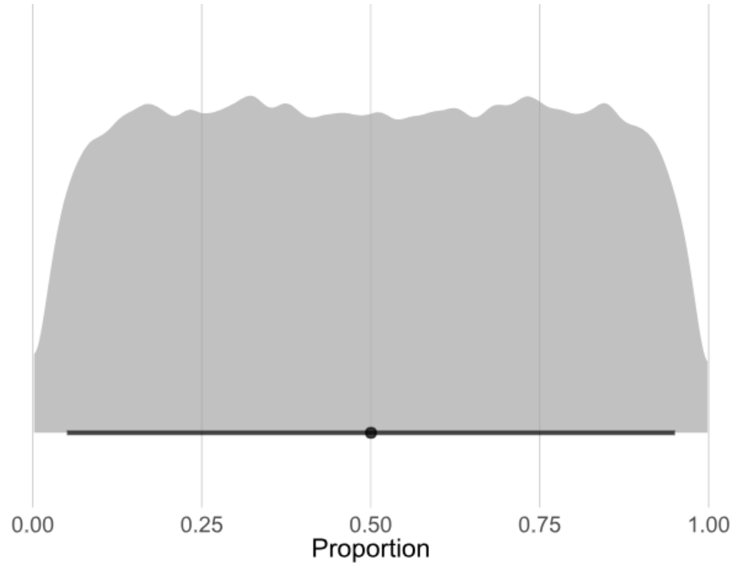

Figure S9. Weakly informative prior for overall effect.

For the between-study heterogeneity parameter ("tau"), we used a weakly informative prior: Half-Normal(1) for the standard deviation of study log-odds (Röver et al., 2021) (Figure S10):

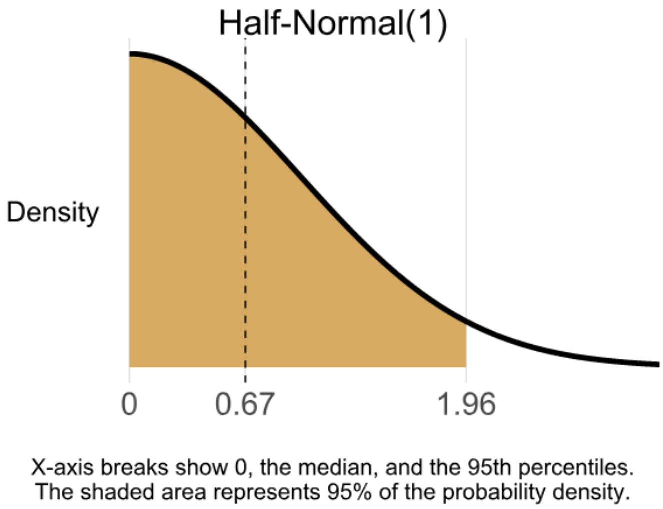

Figure S10. Weakly informative prior Half-Normal (1) for the heterogeneity.

We also did a sensitivity analysis with a vague prior for "tau": Half-Normal(2), while keeping the same weakly informative prior for "mu" (Figure S11):

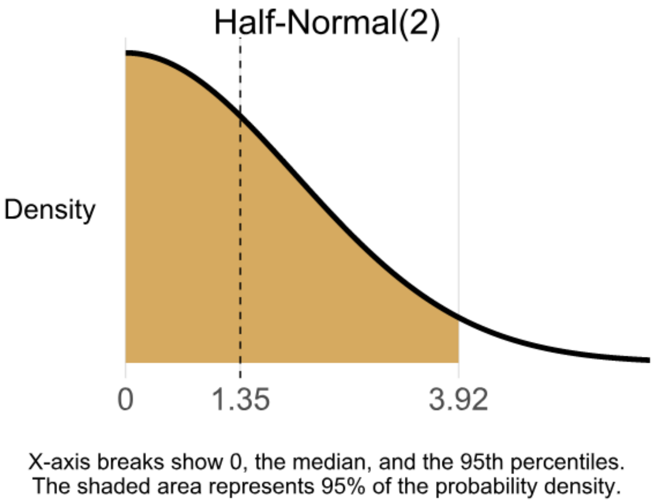

Figure S11. Vague prior Half-Normal (2) for heterogeneity.

MCMC Sampling

The model was fitted using Markov Chain Monte Carlo (Hamiltonian Monte Carlo via Stan) with 4 chains, 2,000 warmup and 2,000 sampling iterations each, and adapt_delta = 0.99.

Subgroup analyses

We conducted Bayesian random-effects meta-regression to explore potential sources of heterogeneity through subgroup analyses. These analyses were performed using the bmr() function from the bayesmeta package, applying the "Intercept/offset" parametrization. In this framework, the first subgroup is defined as the reference (intercept), representing the overall effect within that category (αref), while the remaining coefficients (βn) represent the differences (contrasts) between the subsequent subgroups and the reference.

Prior Specification for Subgroups

Because these are Bayesian meta-regression models, we specified prior distributions for all regression coefficients and the heterogeneity parameter:

- Overall effect in the reference subgroup (αref): We applied a vague prior, Normal(0, 2.72) for binary outcomes (log scale) (Figure S12).

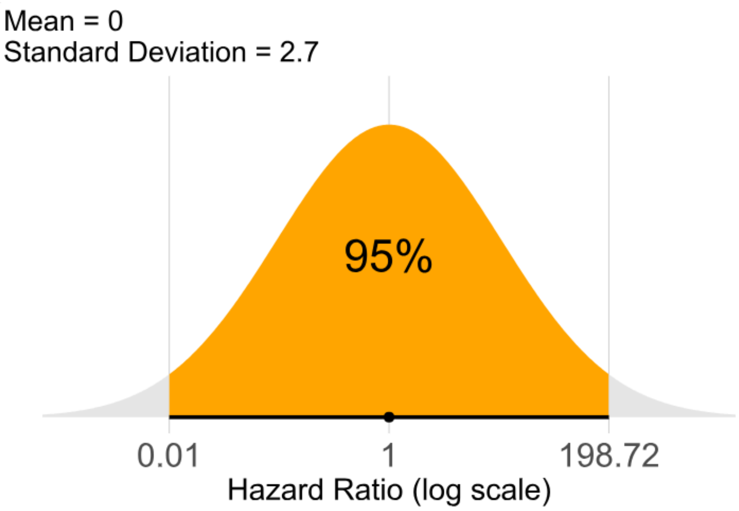

Figure S12. Vague prior distribution for the reference group overall effect**,** using Normal(0, 2.72) for binary outcomes (log scale).

Subgroup differences (βn): We applied a weakly informative prior, assuming that large differences between subgroups are unlikely. We used Normal(0, 0.82) on the log scale, where 95% of the prior density for the Ratio of Hazard Ratios (RHR) ranges from 0.20 to 5.00 (Figure S13), and for proportions we used a weakly informative prior (0, 4.02) (Figure S14).

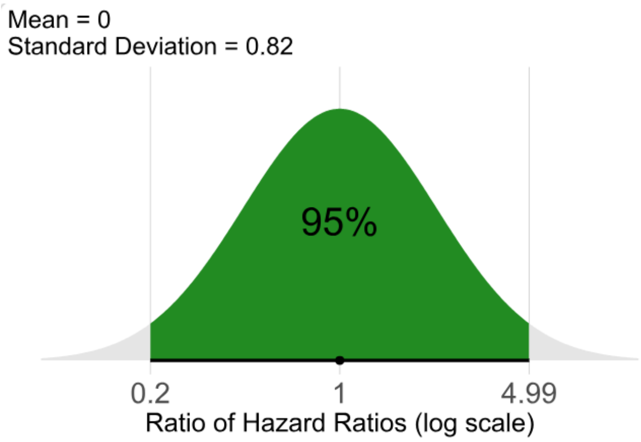

Figure S13. Weakly informative Normal(0, 0.82) prior for subgroup differences on the log scale. The distribution assumes large differences are unlikely, with the 95% density for the Ratio of Hazard Ratios (RHR) ranging from 0.20 to 5.00.

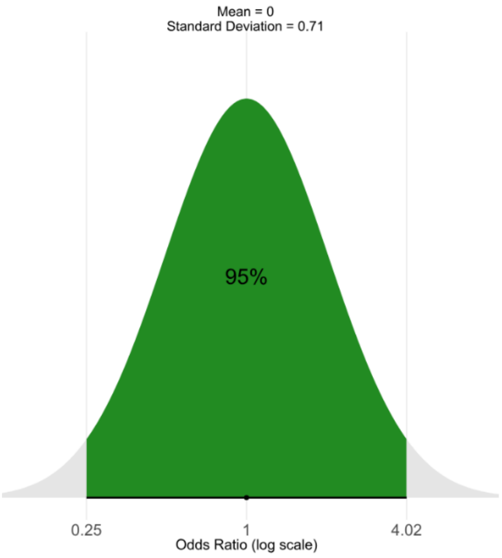

Figure S14. Weakly informative Normal(0, 0.71) prior for subgroup differences on the log scale. The distribution assumes large differences are unlikely, with the 95% density for the proportions ranging from 0.25 to 4.02.

Between-study heterogeneity (τ): The primary analysis for subgroup models used the same informative or weakly informative priors described in the main analysis to ensure consistency in the heterogeneity assumption across groups.

Inference and Visualization

We estimated the marginal posterior distributions for each subgroup and the overall effect using 10,000 posterior samples. To quantify the evidence of a difference between

groups, we calculated the posterior probability that the contrast parameter (β_n) was either greater than or lower than the null value (RHR of 1 for binary). We report the largest probability for each outcome.

We presented the results using forest plots that display the original study estimates alongside the posterior means and 95% CrIs for each subgroup. Additionally, we produced posterior density plots and cumulative distribution plots to visualize the probability of the treatment effect exceeding clinically relevant thresholds across different subgroups.

Sensitivity Analyses

To assess the robustness of our subgroup findings, we conducted sensitivity analyses by varying the prior for the heterogeneity parameter (τ). We compared the primary results with models using a weakly informative Half-Normal(0.5) (Figure S15) and a vague Half-Normal(1.5) prior for binary outcomes (Figure S16). For proportions , we used a weakly informative Half-Normal (1) prior (Figure S17), and a vague Half-Normal (2) prior (Figure 18).

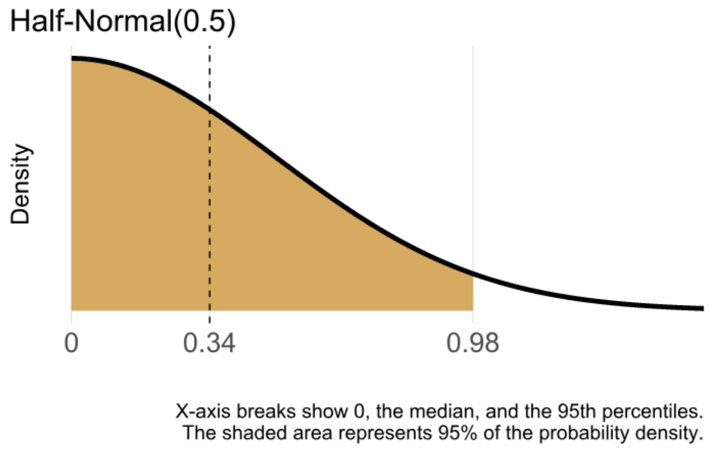

Figure S15. Sensitivity analysis prior for heterogeneity (τ): a weakly informative Half-Normal (0.5) distribution used to assess the robustness of subgroup findings.

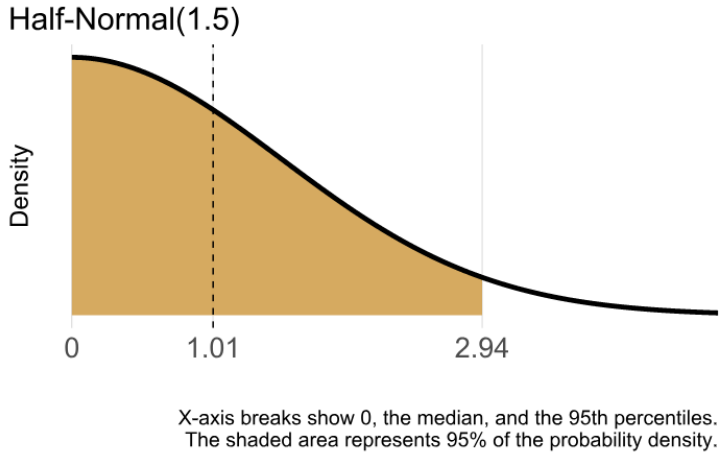

Figure S16. Sensitivity analysis prior for heterogeneity (τ): a weakly informative Half-Normal(1.5) distribution used to assess the robustness of subgroup findings.

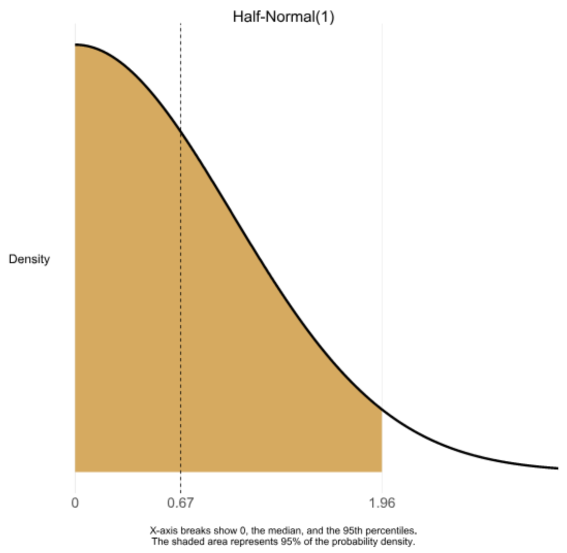

Figure S17. Sensitivity analysis prior for heterogeneity (τ) in proportions: a weakly informative Half-Normal(1) distribution used to assess the robustness of subgroup findings.

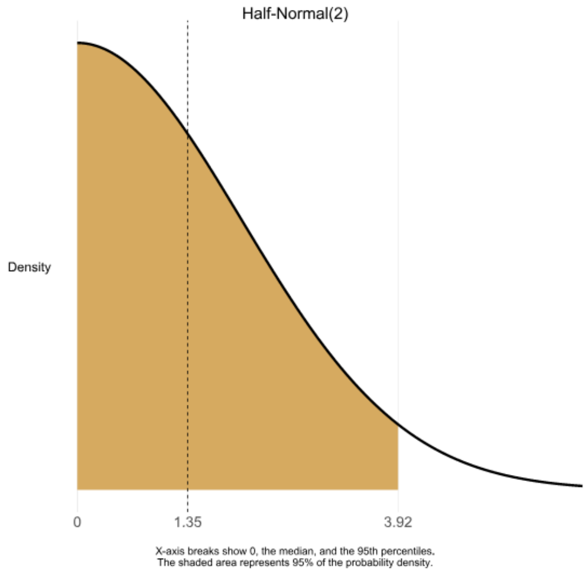

Figure S18. Sensitivity analysis prior for heterogeneity (τ) in proportions: a vague Half-Normal (2) distribution used to assess the robustness of subgroup findings.

Supplementary RESULTS

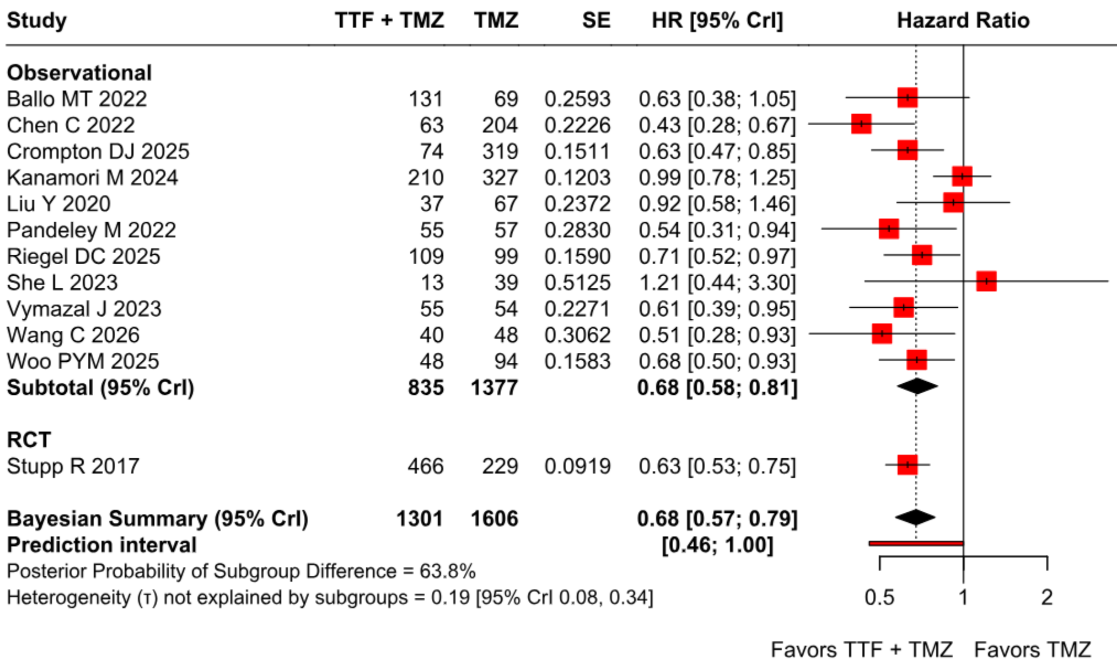

Figure S19. Forest plot showing subgroup analysis of OS by study design. The pooled Hazard Ratio was 0.68 (95% CrI, 0.58–0.81) for observational studies and 0.63 (95% CrI, 0.53–0.75) for RCTs, with a 64% posterior probability of a subgroup difference.

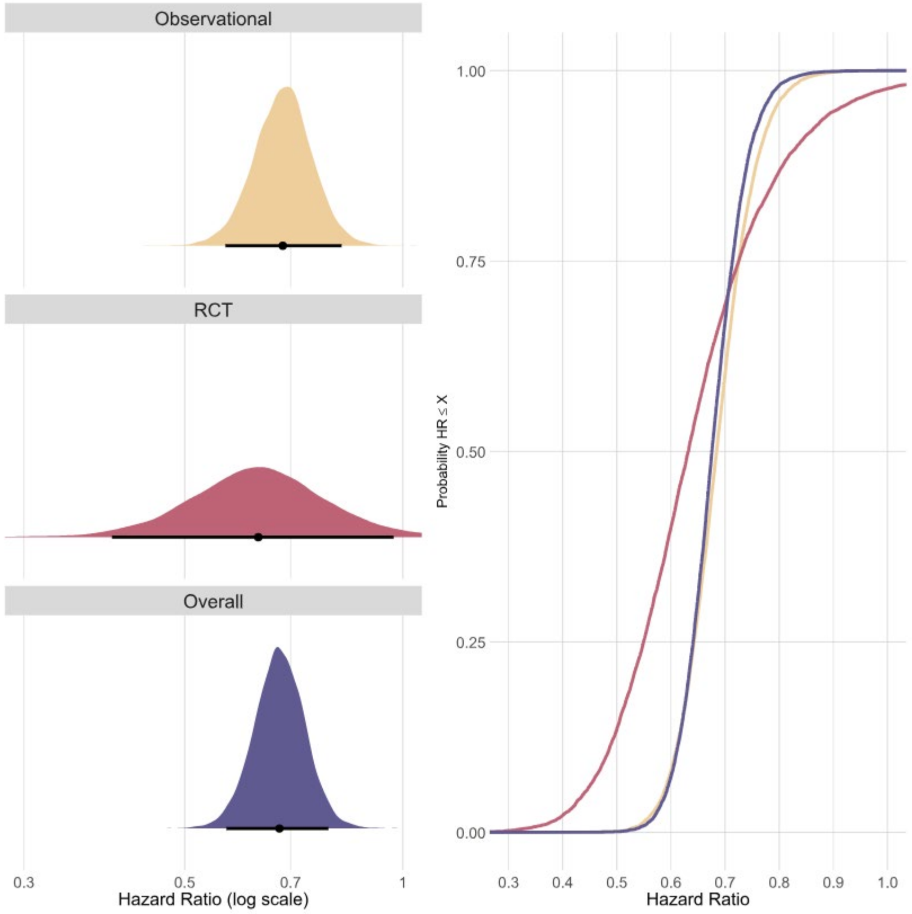

Figure S20. Posterior distributions of subgroup-specific treatment effects. Left panel shows posterior densities for observational studies, RCTs, and overall pooled effect (hazard ratio, log scale). Right panel depicts posterior probabilities that the hazard ratio exceeds 1 across subgroups.

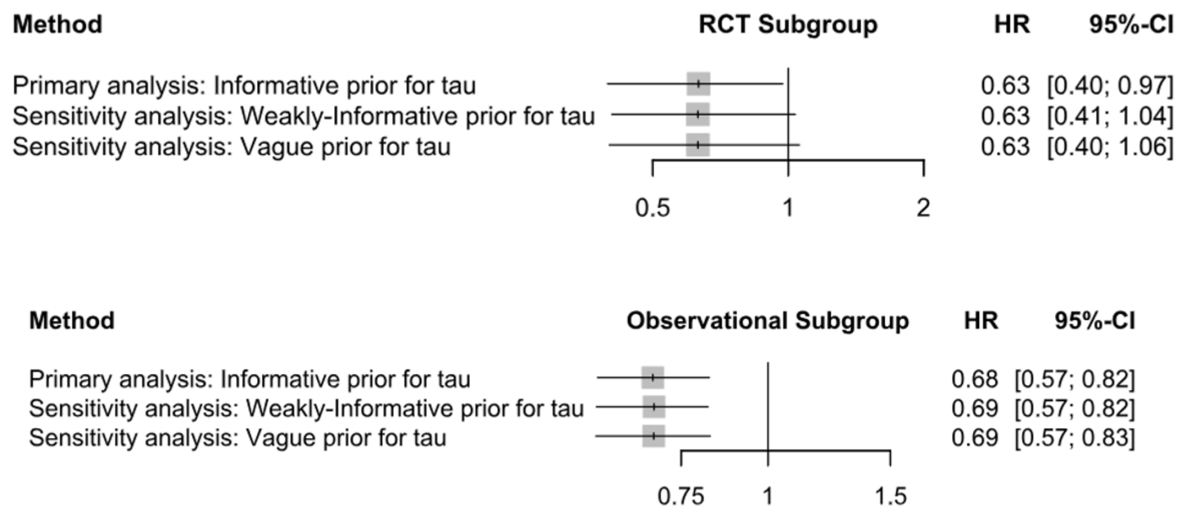

Figure S21. Sensitivity analysis for the observational and RCT subgroup under alternative prior specifications. Forest plot showing posterior hazard ratios and 95% CrI using informative, weakly informative, and vague priors for heterogeneity. Estimates remained directionally consistent across prior assumptions.

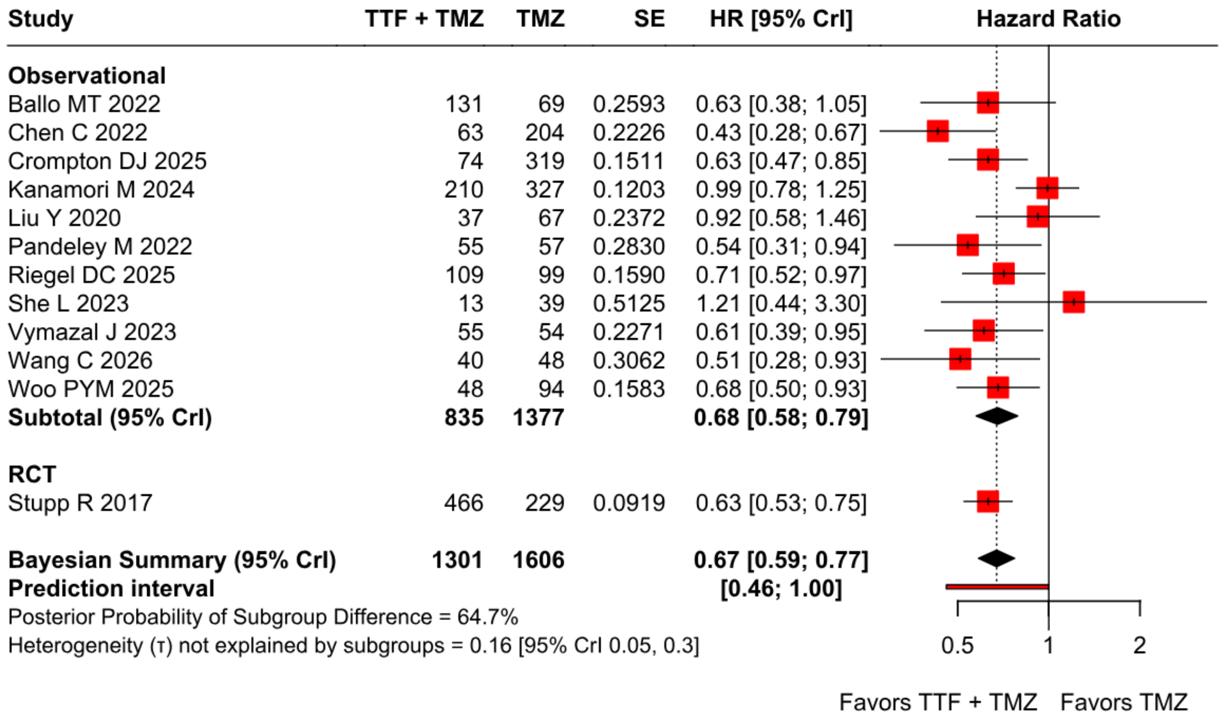

Figure S22. Forest plot showing subgroup analysis of PFS by study design. The pooled Hazard Ratio was 0.68 (95% CrI, 0.58–0.79) for observational studies and 0.63 (95% CrI, 0.53–0.75) for RCTs, with a 64% posterior probability of a subgroup difference.

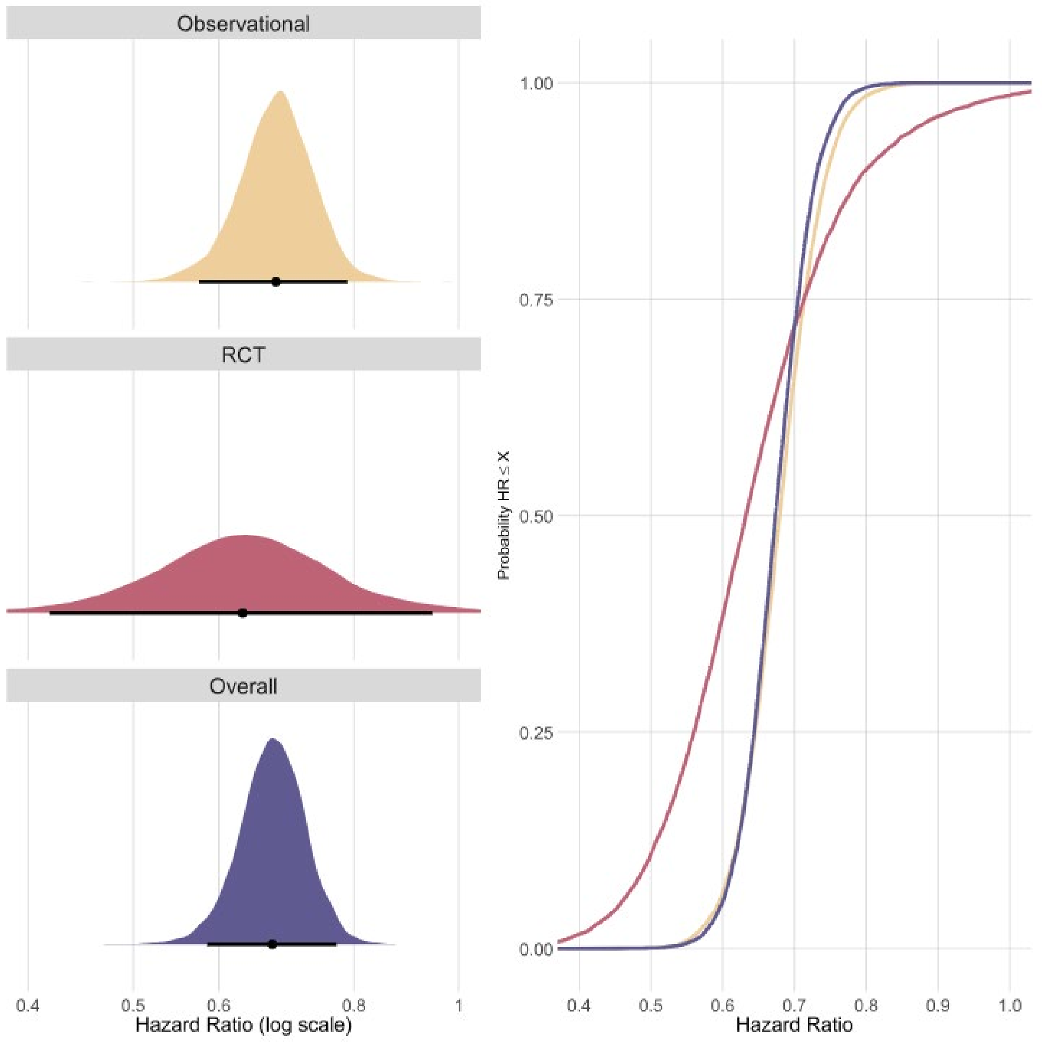

Figure S23. Posterior distributions of subgroup-specific treatment effects. Left panel shows posterior densities for observational studies, RCTs, and overall pooled effect (hazard ratio, log scale). Right panel depicts posterior probabilities that the hazard ratio exceeds 1 across subgroups.

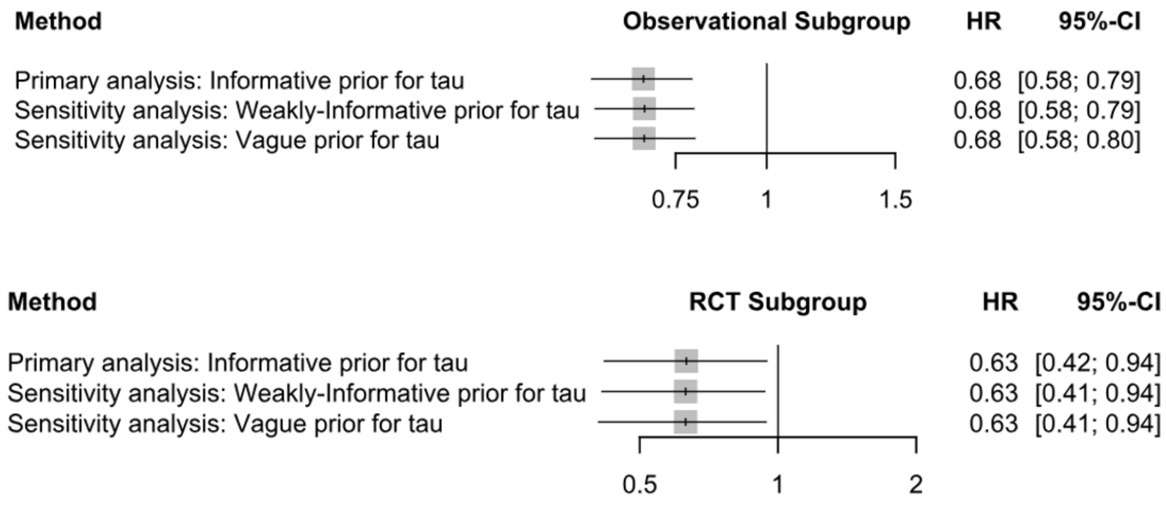

Figure S24. Sensitivity analysis for the observational and RCT subgroup under alternative prior specifications. Forest plot showing posterior hazard ratios and 95% CrI using informative, weakly

informative, and vague priors for heterogeneity. Estimates remained directionally consistent across prior assumptions.

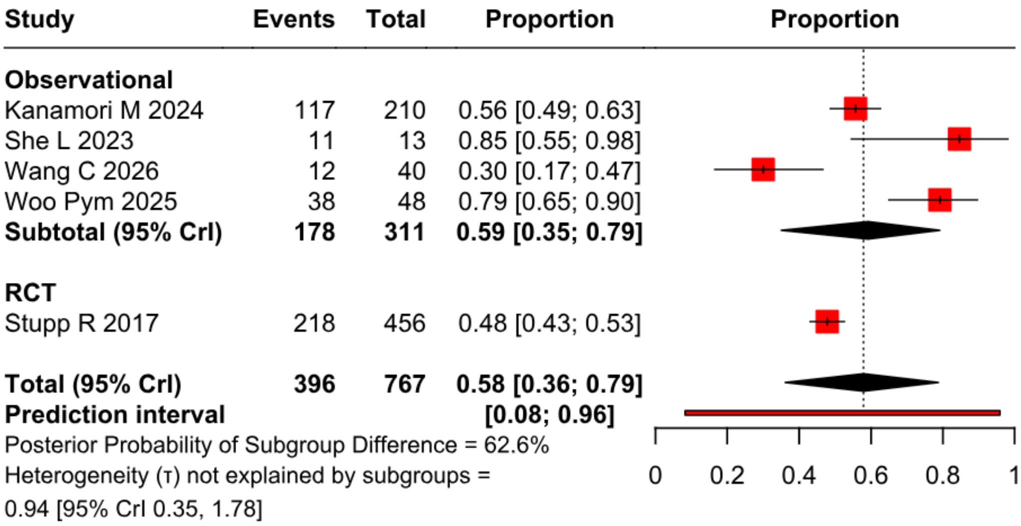

Figure S25. Forest plot showing subgroup analysis of dermatological adverse effects by study design. The pooled proportions were 0.59 (95% CrI, 0.35–0.79) for observational studies and 0.48 (95% CrI, 0.43–0.53) for RCTs, with a 62% posterior probability of a subgroup difference.

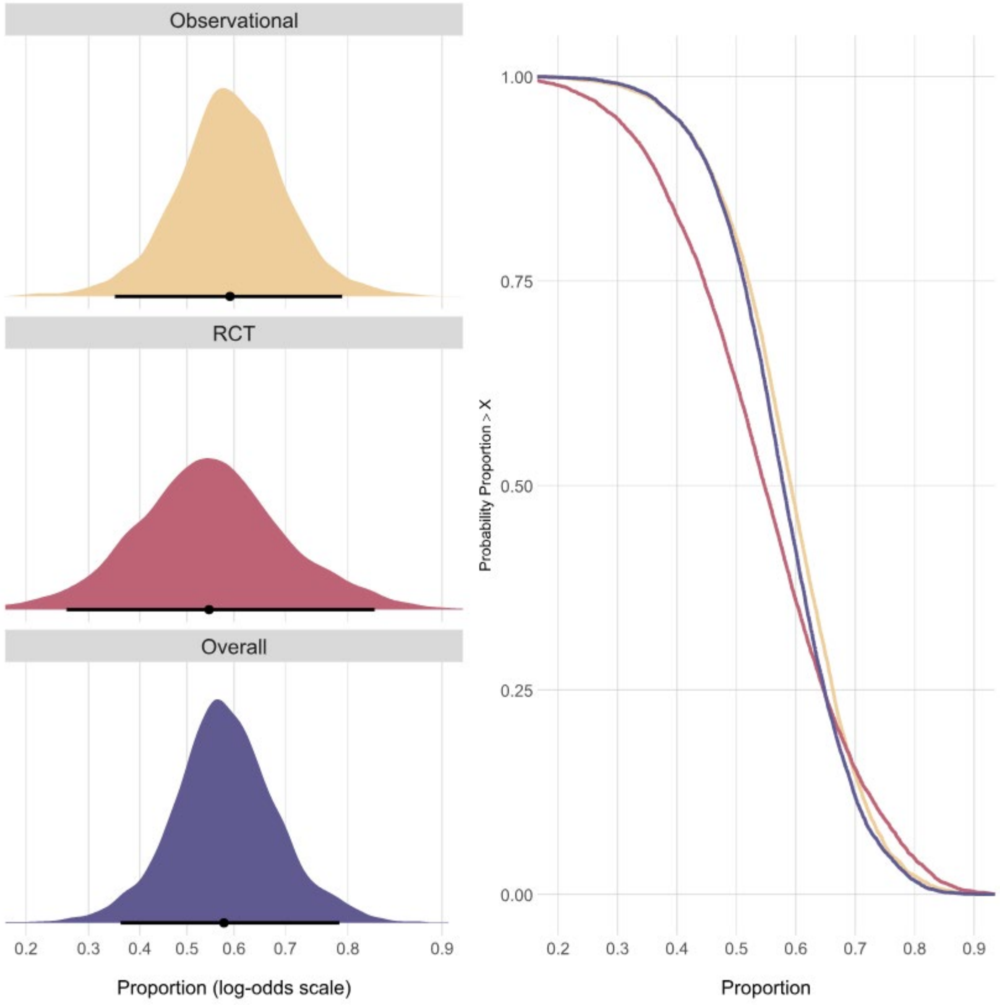

Figure S26. Posterior distributions of subgroup-specific treatment effects. Left panel shows posterior densities for observational studies, RCTs, and overall pooled effect (proportions). Right panel depicts posterior probabilities for harm across subgroups.

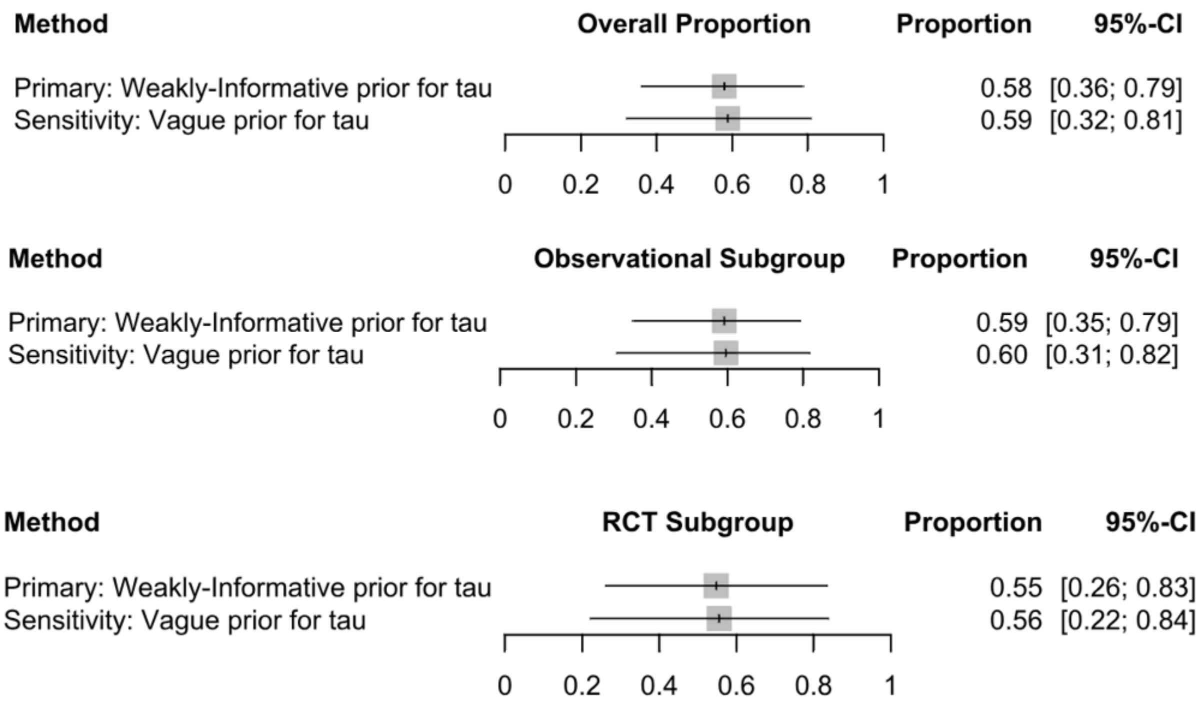

Figure S27. Sensitivity analysis for the observational and RCT subgroup under alternative prior specifications. Forest plot showing posterior proportions and 95% CrI using weakly informative, and vague priors for heterogeneity. Estimates remained directionally consistent across prior assumptions.

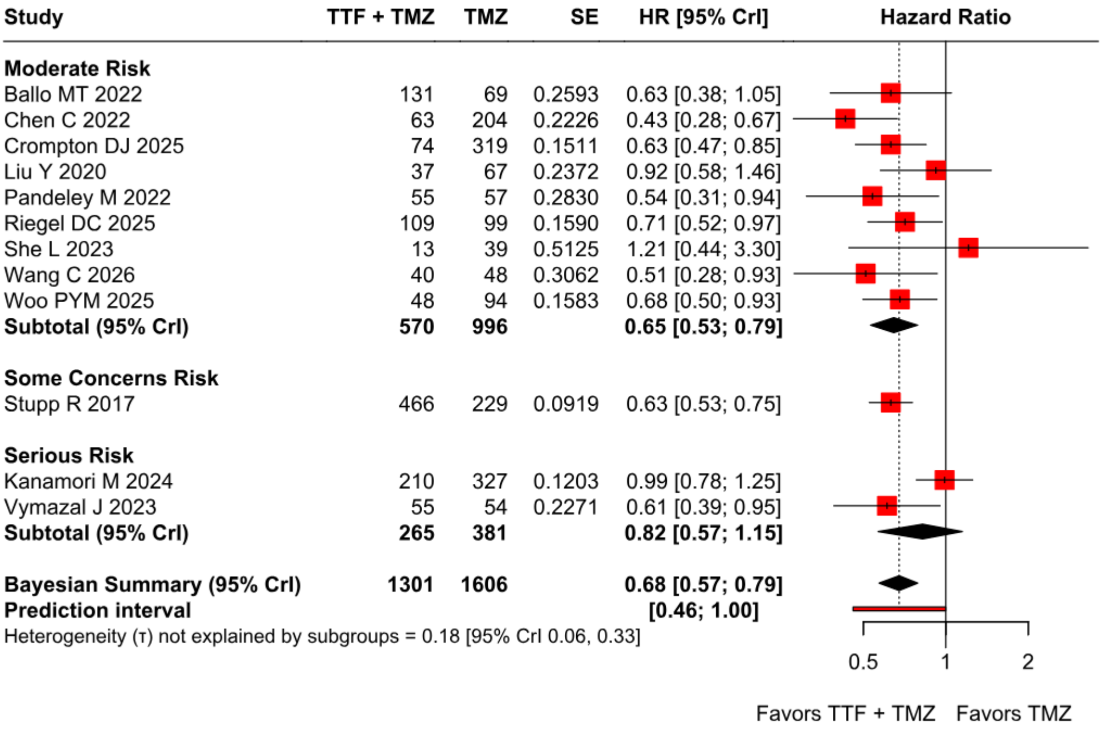

Figure S28. Forest plot showing subgroup analysis of OS by risk of bias. The pooled Hazard Ratio was 0.65 (95% CrI, 0.53–0.79) for moderate risk studies, 0.63 (95% CrI, 0.53-0.75) in some concerns risk studies, and 0.82 (95% CrI, 0.57–1.15) for serious risk.

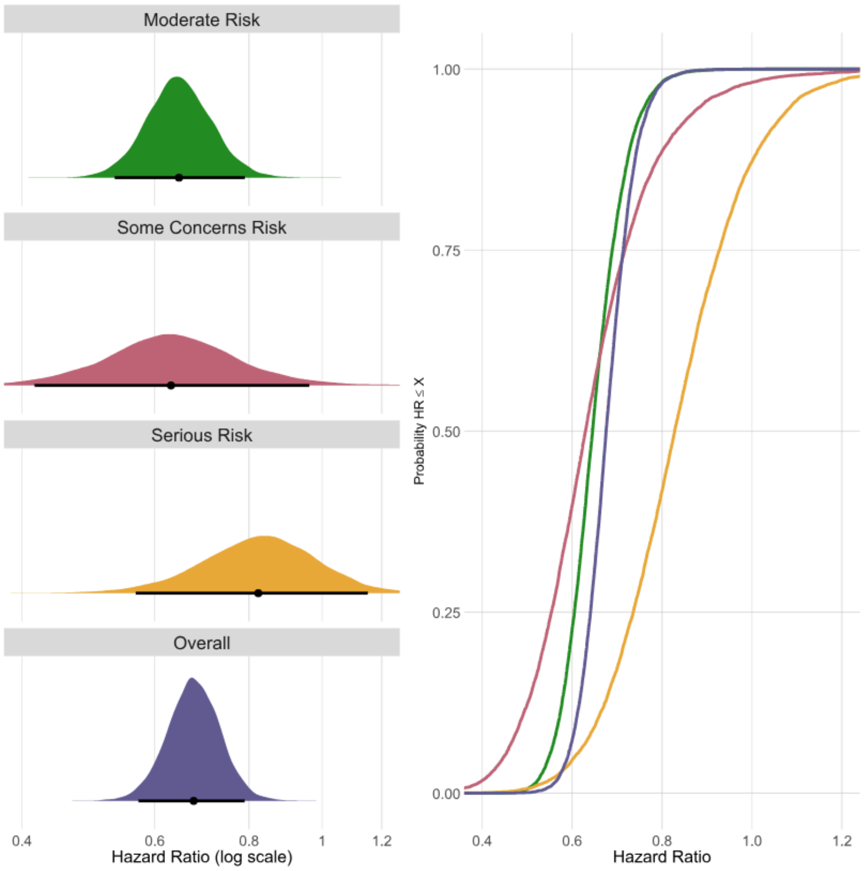

Figure S29. Posterior distributions of subgroup-specific treatment effects. Left panel shows posterior densities for moderate risk, some concerns, serious risk studies, and overall pooled effect (hazard ratios). Right panel depicts posterior probabilities for benefit across subgroups.

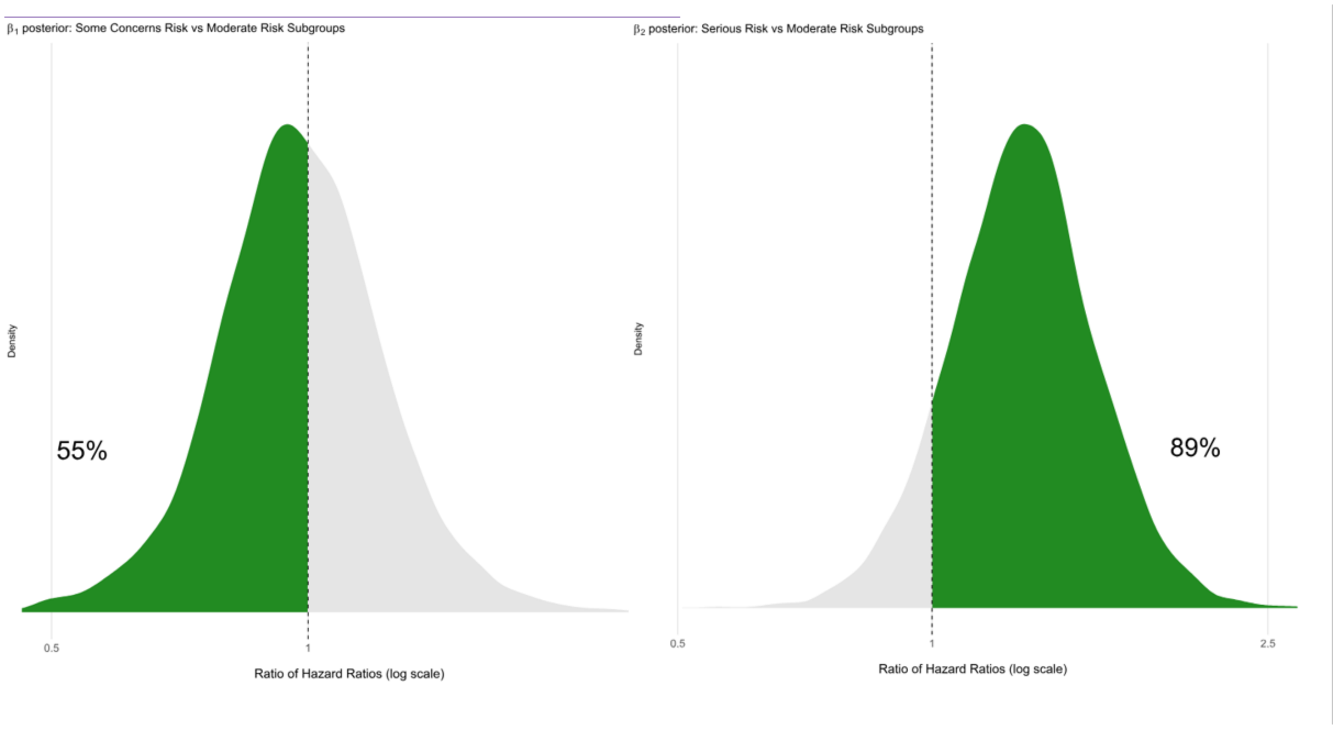

Figure S30. Posterior density plots for pairwise subgroup comparisons. The overall posterior probability of a difference between some concerns and moderate risk studies was 55%, and between serious and moderate risk studies – 89% respectively.

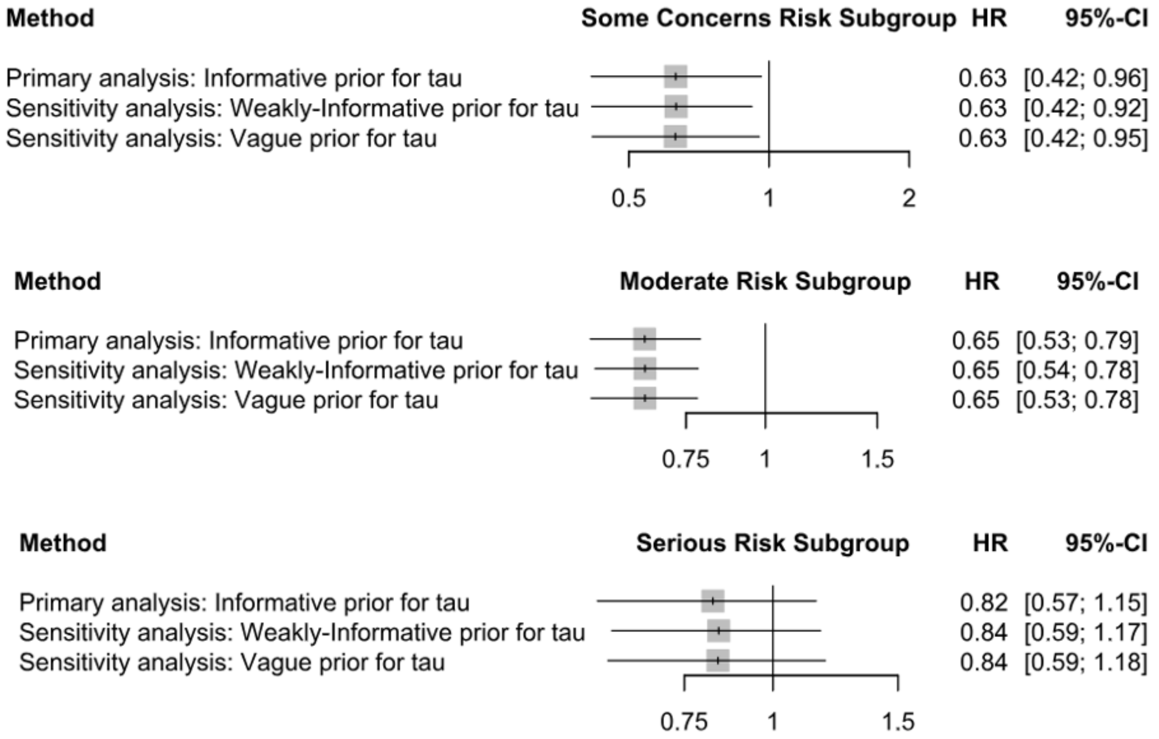

Figure S31. Sensitivity analysis for the some concerns, moderate, and serious risk subgroups under alternative prior specifications. Forest plot showing posterior hazard ratios and 95% CrI using informative, weakly informative, and vague priors for heterogeneity. Estimates remained directionally consistent across prior assumptions.

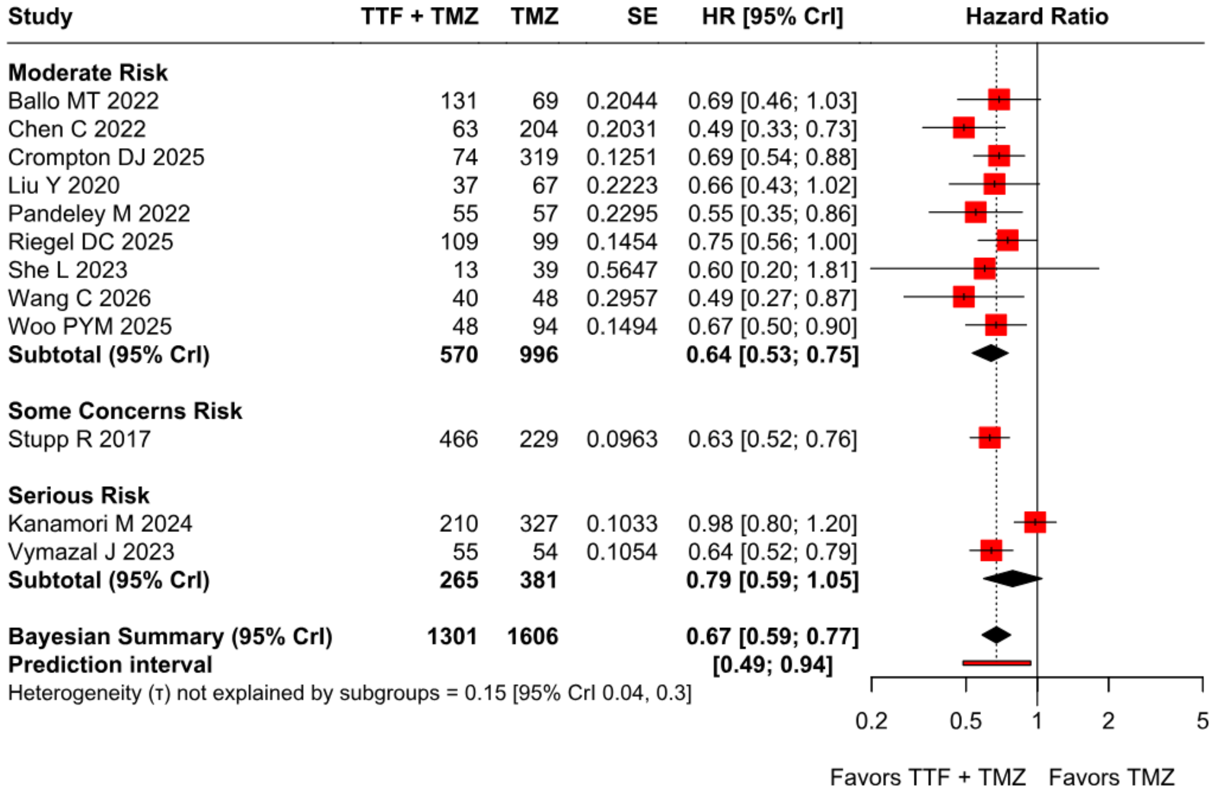

Figure S32. Forest plot showing subgroup analysis of PFS by risk of bias. The pooled Hazard Ratio was 0.64 (95% CrI, 0.53–0.75) for moderate risk studies, 0.63 (95% CrI, 0.52-0.76) in some concerns risk studies, and 0.79 (95% CrI, 0.59–1.05) for serious risk.

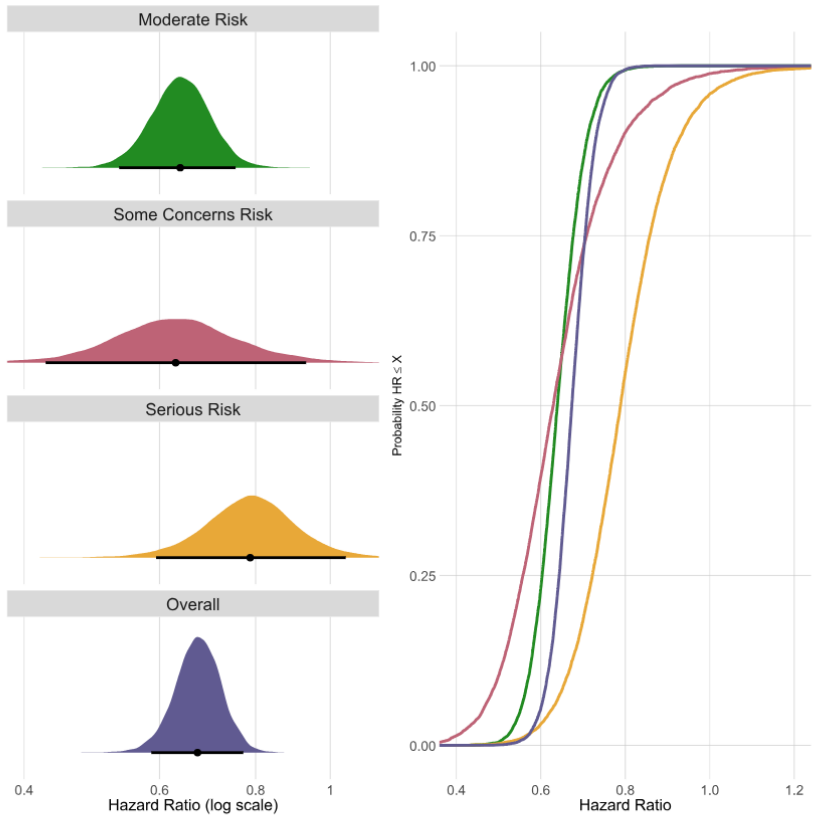

Figure S33. Posterior distributions of subgroup-specific treatment effects. Left panel shows posterior densities for moderate risk, some concerns, serious risk studies, and overall pooled effect (hazard ratios). Right panel depicts posterior probabilities for benefit across subgroups.

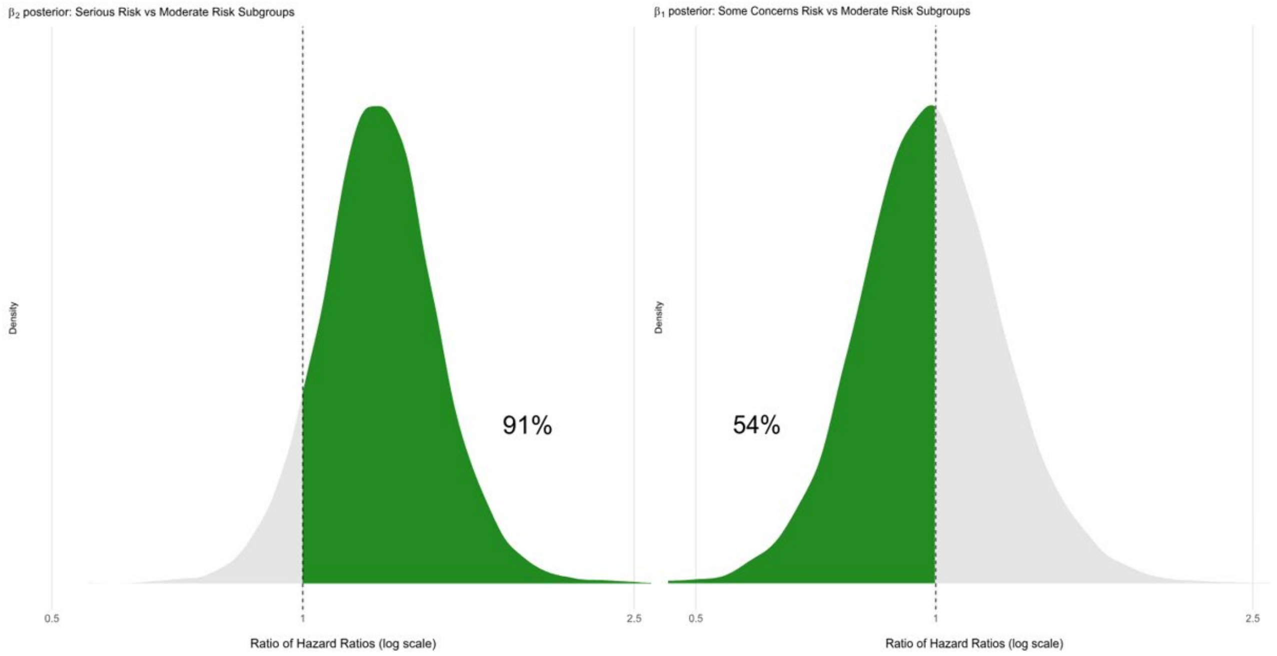

Figure S34. Posterior density plots for pairwise subgroup comparisons. The overall posterior probability of a difference between some concerns and moderate risk studies was 54%, and between serious and moderate risk studies – 91% respectively.

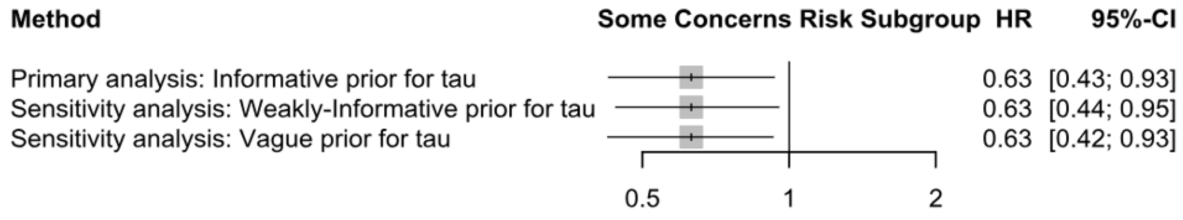

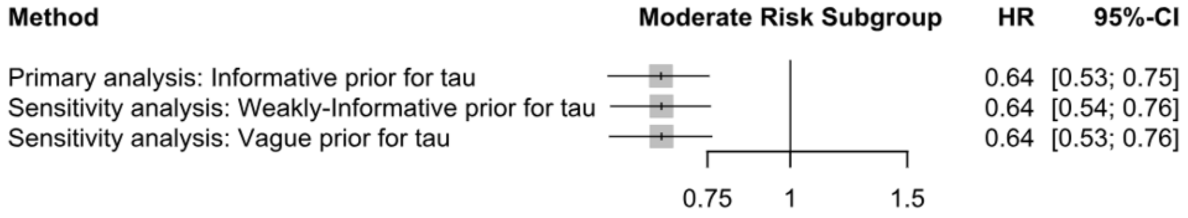

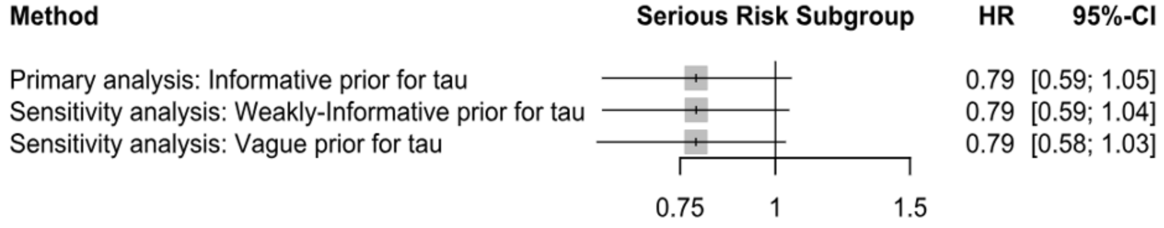

Figure S35. Sensitivity analysis for the some concerns, moderate, and serious risk subgroups under alternative prior specifications. Forest plot showing posterior hazard ratios and 95% CrI using informative, weakly informative, and vague priors for heterogeneity. Estimates remained directionally consistent across prior assumptions.

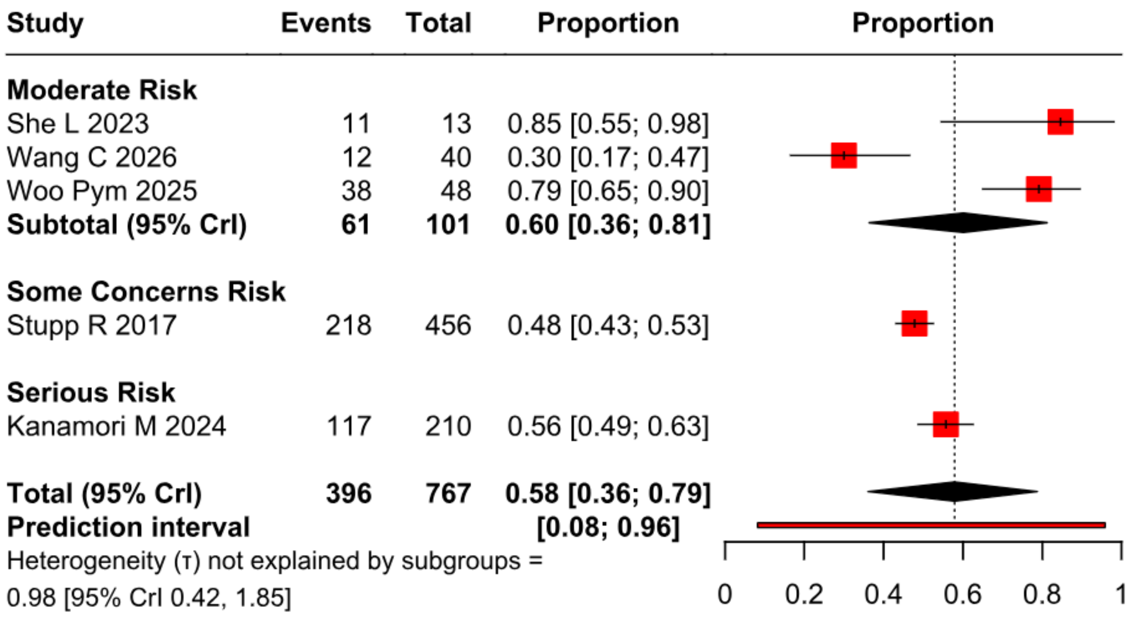

Figure S36. Forest plot showing subgroup analysis of dermatologic adverse effects by risk of bias. The pooled proportion was 0.60 (95% CrI, 0.36–0.81) for moderate risk studies, 0.48 (95% CrI, 0.43-0.53) in some concerns risk studies, and 0.56 (95% CrI, 0.49–0.63) for serious risk.

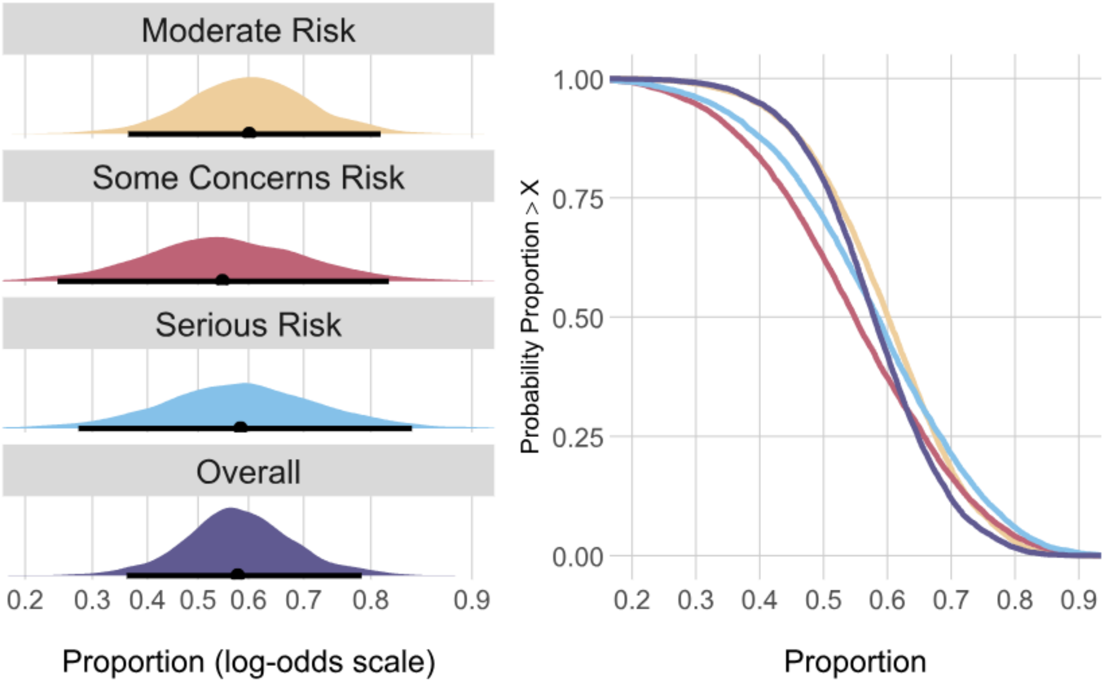

Figure S37. Posterior distributions of subgroup-specific treatment effects. Left panel shows posterior densities for moderate risk, some concerns, serious risk studies, and overall pooled effect (proportions). Right panel depicts posterior probabilities for harm across subgroups.

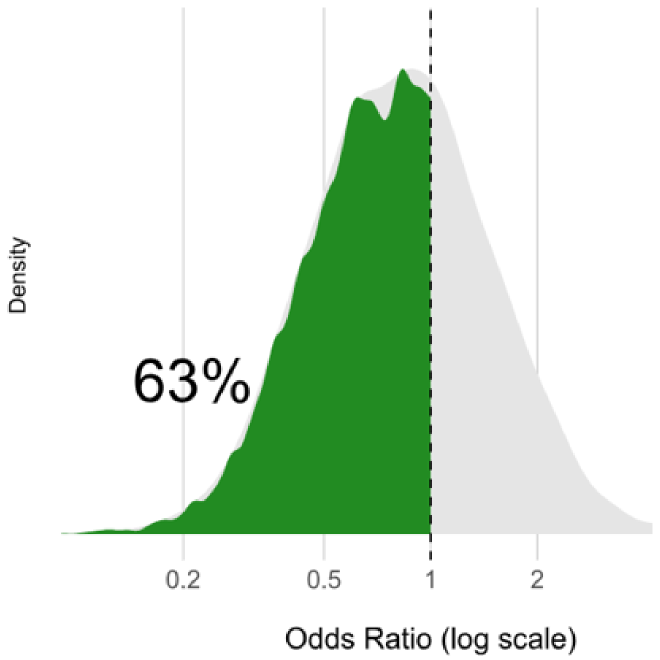

Figure S38. Posterior density plots for pairwise subgroup comparisons. The overall posterior probability of a difference between some concerns and moderate risk studies was 63%, and between serious and moderate risk studies – 55% respectively.

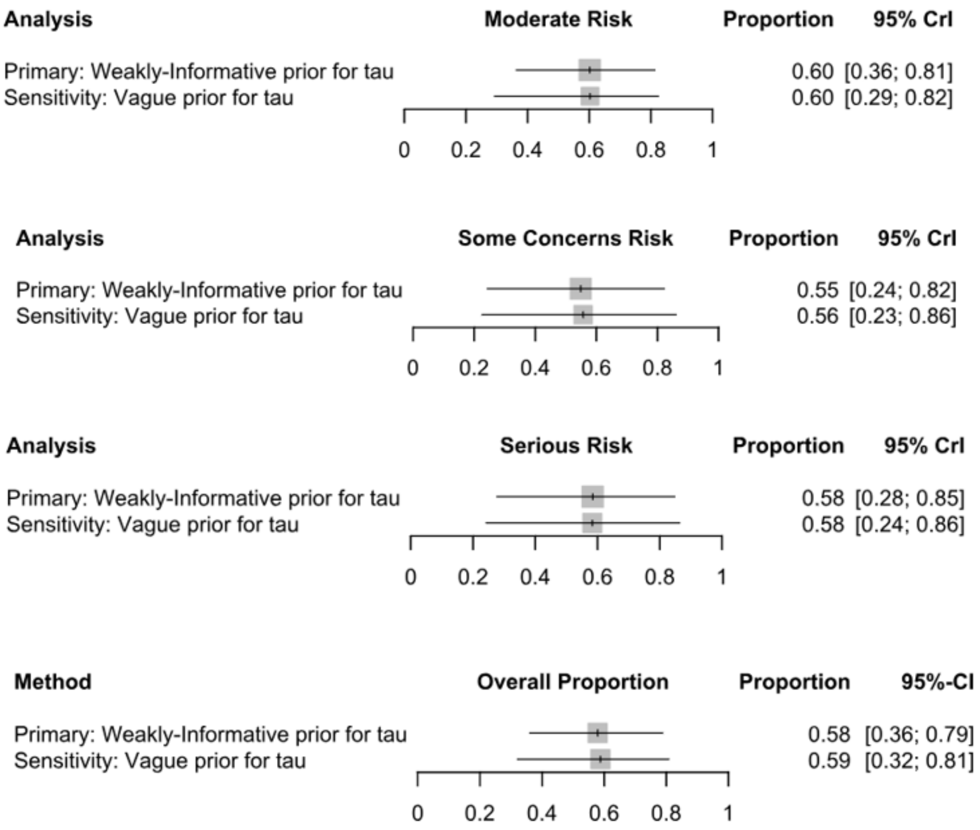

Figure S39. Sensitivity analysis for the some concerns, moderate, and serious risk subgroups under alternative prior specifications. Forest plot showing posterior proportions and 95% CrI using weakly informative, and vague priors for heterogeneity. Estimates remained directionally consistent across prior assumptions.

PRISMA 2020 Checklist

Section and Topic	Item #	Checklist item	Location where item is reported
**TITLE**			
Title	1	Identify the report as a systematic review.	Title, 1-2
**ABSTRACT**			
Abstract	2	See the PRISMA 2020 for Abstracts checklist.	Abstract, 24-45
**INTRODUCTION**			
Rationale	3	Describe the rationale for the review in the context of existing knowledge.	Introduction, 47-78
Objectives	4	Provide an explicit statement of the objective(s) or question(s) the review addresses.	Introduction, 79-83
**METHODS**			
Eligibility criteria	5	Specify the inclusion and exclusion criteria for the review and how studies were grouped for the syntheses.	Methods, 86-104
Information sources	6	Specify all databases, registers, websites, organisations, reference lists and other sources searched or consulted to identify studies. Specify the date when each source was last searched or consulted.	Methods, 106-118
Search strategy	7	Present the full search strategies for all databases, registers and websites, including any filters and limits used.	Methods, 106-118
Selection process	8	Specify the methods used to decide whether a study met the inclusion criteria of the review, including how many reviewers screened each record and each report retrieved, whether they worked independently, and if applicable, details of automation tools used in the process.	Methods, 106-118
Data collection process	9	Specify the methods used to collect data from reports, including how many reviewers collected data from each report, whether they worked independently, any processes for obtaining or confirming data from study investigators, and if applicable, details of automation tools used in the process.	Methods, 106-118
Data items	10a	List and define all outcomes for which data were sought. Specify whether all results that were compatible with each outcome domain in each study were sought (e.g. for all measures, time points, analyses), and if not, the methods used to decide which results to collect.	Methods, 119-122
	10b	List and define all other variables for which data were sought (e.g. participant and intervention characteristics, funding sources). Describe any assumptions made about any missing or unclear information.	Methods, 119-122
Study risk of bias assessment	11	Specify the methods used to assess risk of bias in the included studies, including details of the tool(s) used, how many reviewers assessed each study and whether they worked independently, and if applicable, details of automation tools used in the process.	Methods, 123-132
Effect measures	12	Specify for each outcome the effect measure(s) (e.g. risk ratio, mean difference) used in the synthesis or presentation of results.	Methods, 133-181
Synthesis methods	13a	Describe the processes used to decide which studies were eligible for each synthesis (e.g. tabulating the study intervention characteristics and comparing against the planned groups for each synthesis (item #5)).	Methods, 133-181
	13b	Describe any methods required to prepare the data for presentation or synthesis, such as handling of missing summary statistics, or data conversions.	Methods, 133-181
	13c	Describe any methods used to tabulate or visually display results of individual studies and syntheses.	Methods, 133-181
	13d	Describe any methods used to synthesize results and provide a rationale for the choice(s). If meta-analysis was performed, describe the	Methods,

PRISMA 2020 Checklist

Section and Topic	Item #	Checklist item	Location where item is reported
		model(s), method(s) to identify the presence and extent of statistical heterogeneity, and software package(s) used.	133-181
	13e	Describe any methods used to explore possible causes of heterogeneity among study results (e.g. subgroup analysis, meta-regression).	Methods, 133-181
	13f	Describe any sensitivity analyses conducted to assess robustness of the synthesized results.	Methods, 133-181
Reporting bias assessment	14	Describe any methods used to assess risk of bias due to missing results in a synthesis (arising from reporting biases).	Methods, 123-132
Certainty assessment	15	Describe any methods used to assess certainty (or confidence) in the body of evidence for an outcome.	Not performed
**RESULTS**			
Study selection	16a	Describe the results of the search and selection process, from the number of records identified in the search to the number of studies included in the review, ideally using a flow diagram.	Results, 182-189
	16b	Cite studies that might appear to meet the inclusion criteria, but which were excluded, and explain why they were excluded.	Results, 182-189
Study characteristics	17	Cite each included study and present its characteristics.	Results, 182-189
Risk of bias in studies	18	Present assessments of risk of bias for each included study.	Results, 493-504
Results of individual studies	19	For all outcomes, present, for each study: (a) summary statistics for each group (where appropriate) and (b) an effect estimate and its precision (e.g. confidence/credible interval), ideally using structured tables or plots.	Results, 209-493
Results of syntheses	20a	For each synthesis, briefly summarise the characteristics and risk of bias among contributing studies.	Results, 209-493
	20b	Present results of all statistical syntheses conducted. If meta-analysis was done, present for each the summary estimate and its precision (e.g. confidence/credible interval) and measures of statistical heterogeneity. If comparing groups, describe the direction of the effect.	Results, 209-493
	20c	Present results of all investigations of possible causes of heterogeneity among study results.	Results, 209-493
	20d	Present results of all sensitivity analyses conducted to assess the robustness of the synthesized results.	Results, 209-493
Reporting biases	21	Present assessments of risk of bias due to missing results (arising from reporting biases) for each synthesis assessed.	Results, 493-504
Certainty of evidence	22	Present assessments of certainty (or confidence) in the body of evidence for each outcome assessed.	Not performed
**DISCUSSION**			
Discussion	23a	Provide a general interpretation of the results in the context of other evidence.	Discussion, 557-625
	23b	Discuss any limitations of the evidence included in the review.	Discussion, 633-638

PRISMA 2020 Checklist

Section and Topic	Item #	Checklist item	Location where item is reported
	23c	Discuss any limitations of the review processes used.	Discussion, 633-638
	23d	Discuss implications of the results for practice, policy, and future research.	Discussion, 626-632
**OTHER INFORMATION**			
Registration and protocol	24a	Provide registration information for the review, including register name and registration number, or state that the review was not registered.	Methods, 86-104
	24b	Indicate where the review protocol can be accessed, or state that a protocol was not prepared.	Methods, 86-104
	24c	Describe and explain any amendments to information provided at registration or in the protocol.	Methods, 86-104
Support	25	Describe sources of financial or non-financial support for the review, and the role of the funders or sponsors in the review.	Under references, 855
Competing interests	26	Declare any competing interests of review authors.	Under references, 859
Availability of data, code and other materials	27	Report which of the following are publicly available and where they can be found: template data collection forms; data extracted from included studies; data used for all analyses; analytic code; any other materials used in the review.	Under references, 865

From: Page MJ, McKenzie JE, Bossuyt PM, Boutron I, Hoffmann TC, Mulrow CD, et al. The PRISMA 2020 statement: an updated guideline for reporting systematic reviews. BMJ 2021;372:n71. doi: 10.1136/bmj.n71

For more information, visit: http://www.prisma-statement.org/
